# Deterioration of neuroimmune homeostasis in Alzheimer’s Disease patients who survive a COVID-19 infection

**DOI:** 10.1186/s12974-024-03196-3

**Published:** 2024-08-17

**Authors:** Jonathan A. B. Villareal, Tim Bathe, Gabriela P. Hery, Jennifer L. Phillips, Wangchen Tsering, Stefan Prokop

**Affiliations:** 1 Department of Pathology, Immunology & Laboratory Medicine, University of Florida, Gainesville, FL 32610 USA; 2 Center for Translational Research in Neurodegenerative Disease, University of Florida, Gainesville, FL 32610 USA; 3 Department of Small Animal Clinical Sciences, College of Veterinary Medicine, University of Florida, Gainesville, FL 32608 USA; 4 Department of Neuroscience, University of Florida, Gainesville, FL 32610 USA; 5 Fixel Institute for Neurological Diseases, University of Florida, Gainesville, FL 32608 USA; 6 McKnight Brain Institute, University of Florida, Gainesville, FL 32610 USA

**Keywords:** Microglia, COVID-19, Alzheimer’s disease, Astrocytes, Systemic infection, Neuroinflammation, Oligodendrocytes

## Abstract

Growing evidence has implicated systemic infection as a significant risk factor for the development and advancement of Alzheimer’s disease (AD). With the emergence of SARS-CoV-2 (COVID-19) and the resultant pandemic, many individuals from the same aging population vulnerable to AD suffered a severe systemic infection with potentially unidentified long-term consequences for survivors. To study the impact of COVID-19 survival on the brain’s intrinsic immune system in a population also suffering from AD, we profiled post-mortem brain tissue from patients in the UF Neuromedicine Human Brain and Tissue Bank with a diagnosis of AD who survived a COVID-19 infection (COVID-AD) and contrasted our findings with AD patients who did not experience a COVID-19 infection, including a group of brain donors who passed away before arrival of SARS-CoV-2 in the United States. We assessed disease-relevant protein pathology and microglial and astrocytic markers by quantitative immunohistochemistry and supplemented these data with whole tissue gene expression analysis performed on the NanoString nCounter^®^ platform. COVID-AD patients showed slightly elevated Aβ burden in the entorhinal, fusiform, and inferior temporal cortices compared to non-COVID-AD patients, while tau pathology burden did not differ between groups. Analysis of microglia revealed a significant loss of microglial homeostasis as well as exacerbated microgliosis in COVID-AD patients compared to non-COVID-AD patients in a brain region-dependent manner. Furthermore, COVID-AD patients showed reduced cortical astrocyte numbers, independent of functional subtype. Transcriptomic analysis supported these histological findings and, in addition, identified a dysregulation of oligodendrocyte and myelination pathways in the hippocampus of COVID-AD patients. In summary, our data demonstrate a profound impact of COVID-19 infection on neuroimmune and glial pathways in AD patients persisting for months post-infection, highlighting the importance of peripheral to central neuroimmune crosstalk in neurodegenerative diseases.

## Background

Alzheimer’s disease (AD) is a chronic neurodegenerative disorder that has persisted as one of humanity’s longest-lasting challenges for its aging population. Manifesting in the form of progressive cognitive decline and neurological complications that can eventually lead to death, the brains of individuals suffering from AD are characterized by a compromised neural environment laden with beta-amyloid (Aβ) plaques and hyperphosphorylated tau in the form of neurofibrillary tangles (NFT). Despite the human brain existing as a relatively immune-privileged site within the body, mounting evidence has suggested the involvement of the peripheral and central nervous system (CNS) immunity – as well as the communication between the two - in the pathogenesis of neurodegenerative proteinopathies [1–6]. While current literature on whether neuroimmune involvement accelerates or mitigates AD progression is still highly divisive, it has been suggested that sustained inflammation within the brain parenchyma could make it more vulnerable to degenerative processes [7]. Emerging research has now shifted toward understanding the potential role that peripheral inflammation can play in the development of neurodegenerative diseases such as AD. Systemic infections, defined as infections that are not limited to a single organ or region of the body, can act as sources of peripheral inflammation, with diseases such as influenza, sepsis, and more recently COVID-19 exhibiting body-wide sequelae [8–10].

COVID-19, caused by the SARS-CoV-2 virus and responsible for the recent global pandemic, is of particular interest due to its ubiquitous distribution worldwide and preliminary findings suggesting specific neurological involvement amongst other symptoms [11]. As of March 2024, more than 774 million confirmed cases of COVID-19 have been reported since the emergence of the pathogen, accounting for over 7 million deaths and equivalent to almost 10% of the world’s population having experienced systemic infection via SARS-CoV-2, not including unreported cases [12]. COVID-19 has presented with various neurological sequelae, with reports of persistent ‘brain fog’ and cognitive impairment months post-infection in the absence of sustained viral positivity (RNA and protein) in the brain [11, 13, 14]. These findings are suggestive of the ability of peripheral systemic infections (i.e. lungs in COVID-19) to communicate chronic damage to the brain in the absence of direct neuroinvasion and persistence, potentially through inflammation-mediated pathways. Within the CNS, microglia act as the main immune-modulating cell type and the resident macrophages of the parenchyma, with powerful cytokine-generating and phagocytotic functions [15]. Neuropathological studies have shown that elderly, non-AD patients who have an active and severe COVID-19 infection at the time of death present with heightened microgliosis and microglial activation [16]. Similarly, patients with end-stage AD have been observed to possess dysregulated microglial homeostasis, with augmented microgliosis and chronically activated phenotypes being shared between COVID-19 and AD [17–20]. Considering the significant overlap between the vulnerable age ranges for these diseases (65 + for both COVID-19 and AD) [21, 22], there remains an important gap in knowledge concerning the intersection of these two diseases and the interactions between their respective immunomodulatory effects.

To evaluate the potential effects of surviving a systemic infection on AD pathophysiology, we performed immunohistochemical (IHC) profiling of microglia from individuals with a post-mortem AD diagnosis who either reported a COVID-19 infection during their lives (COVID) or did not (non-COVID). We also extended this analysis to astrocytes due to their accessory roles in neuroimmunity [23–26] as well as their role in the formation of the blood-brain barrier (BBB) [27, 28], both of which have been implicated to be involved in AD and COVID-19 outcomes. Previous studies have identified the choroid plexus (CP) as a primary interface for communicating inflammation between the periphery and the CNS during active COVID-19 infection [14, 29, 30], making CP-adjacent regions such as the hippocampus more susceptible to immune augmentation. As microglial activation in AD shows brain region-specific patterns [31], we decided to evaluate these immune interactions across 4 locations in the brain, taking both AD staging and CP proximity into account. Additionally, we performed whole tissue transcriptomics using the NanoString nCounter^®^ platform to evaluate gene expression profiles of glial cells in COVID-AD patients and to identify other novel changes potentially associated with co-occurrence of COVID-19 and AD.

## Methods and materials

### Patient tissue samples and cohort selection

**Fig. 1:**
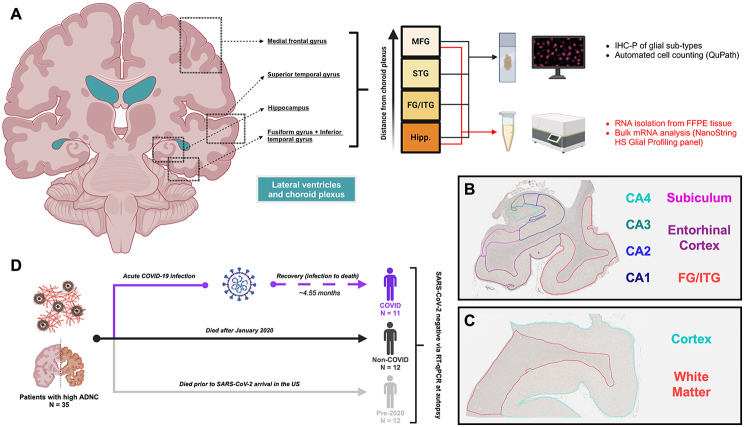
Cohort overview and regional differences in COVID-AD. (**A**) Four regions of interest (MFG, STG, Hippocampus (Hipp.), and FG/ITG) were analyzed in the brains of each patient. Regions were selected with respect to a combination of regional differences in AD progression and CP proximity. Microglial profiling was performed on all four regions and bulk mRNA analysis was performed on the regions nearest and furthest to the CP (hippocampus and medial frontal gyrus, respectively). (**B**) For glial profiling, hippocampal sections were segmented by annotation into the four CA subfields (CA4-CA1), the subiculum, and the entorhinal cortex. The FG/ITG region was attached to hippocampal sections and separated by annotation for analysis. (**C**) The MFG and STG regions were separated by annotation into cortex and white matter for glial profiling. (**D**) All cohort patients selected (*N* = 35) possessed high ADNC at autopsy as defined in the methods. Patients were then separated into groups based on if they suffered from and survived a COVID-19 infection (COVID, *N* = 11), did not experience a COVID-19 infection and died during the same time period as COVID patients (Non-COVID, *N* = 12), or died prior to SARS-CoV-2 arrival in the US on January 20, 2020 (Pre-2020, *N* = 12). Figures were created at BioRender.com.

Human post-mortem tissue was obtained from the University of Florida Human Brain and Tissue Bank following approval by the UF Institutional Review Board. Alzheimer’s Disease neuropathological changes (ADNC) and AD staging for each patient were evaluated by a board-certified neuropathologist, and all patients selected had a primary diagnosis of AD with “high” ADNC based on current NIA-AA guidelines for pathological diagnosis [32, 33] (Table 1). Co-pathology in the form of Lewy body disease LBD, Limbic-predominant age-related TDP-43 proteinopathy (LATE), Aging-related tau astrogliopathy (ARTAG), cerebral amyloid angiopathy (CAA) was classified according to current guidelines [32–38] and as detailed in previous articles from our group [31, 39] (Table 2). AD patients were grouped based on whether they died prior to SARS-CoV-2 arrival in the United States (Pre-2020), did not have a medical history of COVID-19 infection (Non-COVID), or had one or more self-reported COVID-19 infections that were resolved or did not result in death (COVID) (Table 1) (Fig. 1D). The Pre-2020 group was included to assess the possibility of asymptomatic COVID-19 infection (as opposed to severe and reported infection) in the Non-COVID group and to compare it to individuals who could not have had COVID-19 at any point in their lives. Post-infection interval for COVID patients was calculated as the period between reported infection and time of death (Tables 1, and 2).

**Table 1:** Cohort group demographics

	Pre-2020	Non-COVID	COVID
N	12	12	11
Mean age	77.8	78.2	79.6
Sex M/F	6/6	4/8	5/6
Mean Thal staging	4.83	5	4.82
Mean Braak staging	5.25	5.25	5.55
Mean post-mortem interval	14.8	38	36.6
Mean post-infection interval	-	-	4.55 months

**Table 2:** Individual patient demographics

Patient	Cohort Group	Neuropathalogical Diagnosis	Sex	Age (Years)	Thal	Braak	CERAD	Co-Pathologies	Post-mortem Interval (Hours)	Post-Infection Interval (Months)	ApoE	DV200 (%)
1	Covid	AD High	f	79	4	V	frequent	CAA moderate, Diffuse acute SAH and IVH	39.5	5	2/3	50
2	Covid	AD High	f	76	5	VI	frequent	CAA mild, LATE NC stage 2, hippocampal sclerosis	11	1	3/4	77
3	Covid	AD High	m	78	5	VI	frequent	CAA moderate	35	13	4/4	58
4	Covid	AD High	m	83	5	V	frequent	CAA widespread, moderate	34	1	4/4	68
5	Covid	AD High	m	71	4	V	frequent	CAA moderate	46	1	3/3	77
6	Covid	AD High	f	85	5	V	frequent	CAA mild	15	8	3/4	66
7	Covid	AD High	m	94	5	V	frequent	CAA moderate, LBD limbic-transitional, Remote microinfarct in cerebellum	6 days	2	3/3	80
8	Covid	AD High	f	62	5	VI	frequent	CAA moderate, LBD amygdala predominant	36	5	3/3	76
9	Covid	AD High	f	77	5	VI	frequent	CAA moderate, LATE stage 2, hippocampal sclerosis, LBD (amygdala predominant)	9	11	3/3	80
10	Covid	AD High	f	80	5	VI	frequent	CAA severe	19	1	3/3	74
11	Covid	AD High	m	91	5	VI	frequent	CAA severe, LBD diffuse neocortical, LATE stage 1	14.25	2	4/4	62
12	Non-Covid	AD High	f	73	5	V	frequent	CAA severe	3 days		3/4	71
13	Non-Covid	AD High	f	80	5	V	frequent	CAA moderate	34		3/4	78
14	Non-Covid	AD High	f	80	5	V	frequent	CAA mild	5 days		3/4	91
15	Non-Covid	AD High	f	83	5	V	frequent	CAA mild	24		3/3	54
16	Non-Covid	AD High	f	71	5	V	frequent	CAA moderate, LATE stage 1	20		4/4	76
17	Non-Covid	AD High	m	80	5	V	frequent	CAA severe	15		3/4	80
18	Non-Covid	AD High	m	77	5	V	frequent	CAA moderate, LATE stage 2, hippocampal sclerosis	23		3/4	78
19	Non-Covid	AD High	m	78	5	VI	frequent	CAA mild, LBD diffuse neocortical	22		3/4	81
20	Non-Covid	AD High	f	88	5	V	frequent	CAA moderate, LBD amygdala predominant	4 days		2/3	73
21	Non-Covid	AD High	f	83	5	V	frequent	CAA moderate	9		3/3	78
22	Non-Covid	AD High	f	76	5	VI	frequent	None reported	3.5		3/3	75
23	Non-Covid	AD High	m	69	5	VI	frequent	CAA moderate, LBD amygdala predominant	18		3/4	76
24	Pre-2020	AD High	m	73	5	V	frequent	CAA moderate			3/4	
25	Pre-2020	AD High	f	90	5	V	frequent	CAA mild, LATE stage 1			3/3	
26	Pre-2020	AD High	f	82	5	V	frequent	CAA mild, LATE stage 1				
27	Pre-2020	AD High	m	84	4	V	frequent	CAA moderate	16		3/4	
28	Pre-2020	AD High	f	72	5	V	frequent	CAA severe	12		3/4	
29	Pre-2020	AD High	f	78	5	V	frequent	CAA moderate	5		3/3	
30	Pre-2020	AD High	f	78	4	V	frequent	CAA moderate, LATE stage 2	20		3/3	
31	Pre-2020	AD High	m	78	5	V	frequent	CAA mild	20		3/4	
32	Pre-2020	AD High	f	86	5	V	frequent	CAA moderate	14		3/4	
33	Pre-2020	AD High	m	60	5	VI	frequent	CAA severe	20		3/3	
34	Pre-2020	AD High	m	70	5	VI	frequent	CAA moderate	8		4/4	
35	Pre-2020	AD High	m	83	5	VI	frequent	CAA moderate	18		3/3	

### Immunohistochemistry

Glial and protein IHC profiling was performed on either 5–10 μm thick formalin-fixed paraffin-embedded (FFPE) sections depending on the targeted protein (Table 3). Staining was performed as previously described by our laboratory [31] and specific pretreatment protocols for each antibody are summarized below (Table 3). Additionally, sections stained for Olig2 required agitation on an orbital shaker (OHAUS, tilt and speed setting 2) during overnight primary and secondary antibody incubations to produce an adequate signal. GFAP and ALDH1L1 stains were performed with ImmPRESS HRP (Vector Labs) of their respective species added to the goat anti-rabbit/mouse IgG HRP conjugated secondary antibody (Millipore Sigma) at a dilution of 1:1000.

Immunofluorescent double-stain analysis of CD31 and GFAP was performed on 5 μm thick FFPE sections following a majority of the same steps as described above. GFAP was developed in the Texas Red channel with a standard, directly conjugated fluorescent secondary antibody, and CD31 in the FITC channel using ImmPRESS HRP (Vector Labs) in combination with Opal HRP fluorophores (Akoya Biosciences, USA). Sections were mounted using VECTASHIELD HardSet Antifade Mounting Medium with DAPI (Vector Labs) and stored in a dark chamber at 4 °C for long-term storage. Further detail into the immunofluorescent staining procedure can be found below (TableS 3, 4 and 5).

**Table 3:** IHC antibodies and pretreatments

Primary Antibody	Manufacturer	Concerntration	Section Thickness	Antigen Retrieval Solution	Primary Incubation Period	Secondary Antibody	Secondary Incubation Period	Additional notes
Ab5	In-house	1:5000	5 µm	Tris and 0.05% Tween	Overnight @ 4°C	Vector Laboratories ImmPRESS®	30 minutes @ room temperature	Described in Levites et al. 2006 [49]
7F2	In-house	1:5000	5 µm	Tris and 0.05% Tween	Overnight @ 4°C	Vector Laboratories ImmPRESS®	30 minutes @ room temperature	Described in Xia et al., 2020 [50]
Iba1	Abcam - ab178847	1:8000	10 µm	Citrate	Overnight @ 4°C	Vector Laboratories ImmPRESS®	30 minutes @ room temperature	
P2RY12	Millipore Sigma - HPA014518	1:1000	10 µm	Tris and 0.05% Tween	Overnight @ 4°C	Vector Laboratories ImmPRESS®	30 minutes @ room temperature	
CD68	Thermo Fisher - TA807199	1:500	10 µm	Citrate	Overnight @ 4°C	Vector Laboratories ImmPRESS®	30 minutes @ room temperature	
Ferritin (light and heavy chains)	Millipore Sigma - F6136-1MG	1:1000	10 µm	Tris and 0.05% Tween	Overnight @ 4°C	Vector Laboratories ImmPRESS®	30 minutes @ room temperature	
GFAP	Encore - MCA-3E10	1:1000	5 µm	Tris and 0.05% Tween	Overnight @ 4°C	Vector Laboratories - BA-9200-1.5 @ 1:1000	1 hour @ room temperature	Add ImmPRESS® @ 1:1000 to secondary antibody solution
ALDH1L1	Thermo Fisher - UM500039	1:400	5 µm	Tris and 0.05% Tween	Overnight @ 4°C	Vector Laboratories - BA-9200-1.5 @ 1:1000	1 hour @ room temperature	Add ImmPRESS® @ 1:1000 to secondary antibody solution
Olig2	Abcam - ab109186	1:100	5 µm	Citrate	Overnight @ 4°C	Vector Laboratories ImmPRESS®	30 minutes @ room temperature	Agitate on shaker during overnight primary and secondary incubations
PLP1	MCAB396	1:1000	5 µm	Citrate	Overnight @ 4°C	Vector Laboratories ImmPRESS®	30 minutes @ room temperature	
CD3e	Invitrogen - MA5-14524	1:500	5 µm	Citrate	Overnight @ 4°C	Vector Laboratories ImmPRESS®	30 minutes @ room temperature	
GFAP (IF)	Encore - MCA-3E10	1:1000	5 µm	Citrate	Overnight @ 4°C	Invitrogen Anti-mouse DyLight 594 conjugated (35510)		
CD31 (IF)	Proteintech − 11265-1-AP	1:500	5 µm	Citrate	Overnight @ 4°C	Vector Laboratories ImmPRESS®	30 minutes @ room temperature	Develop with Opal 520 @ 1:1000 for 10 minutes

**Table 4:** Top 25 differentially expressed genes in COVID vs. Non-COVID in Hippocampus

	Log2 fold change	std error (log2)	Lower confidence limit (log2)	Upper confidence limit (log2)	Linear fold change	Lower confidence limit (linear)	Upper confidence limit (linear)	P-value	BY.p.value	Method	Gene.sets	probe.ID
GAL3ST1-mRNA	-0.888	0.159	-1.2	-0.576	0.54	0.435	0.671	1.58E-05	0.0579	lm.nb	Myelogenesis	NM_004861.1:1378
TICAM1-mRNA	0.646	0.121	0.41	0.883	1.57	1.33	1.84	2.56E-05	0.0579	lm.nb	Apoptosis, NF-kappaB Signaling, NO Metabolism and Signaling	NM_014261.1:518
CHI3L1-mRNA	2.75	0.563	1.65	3.85	6.72	3.13	14.5	7.91E-05	0.119	lm.nb	Astrocyte Markers	NM_001276.2:475
HTR5A-mRNA	1.46	0.319	0.83	2.08	2.74	1.78	4.23	0.00017	0.155	lm.nb	Calcium Signaling, Neuroactive Ligands and Receptors, Serotonergic Synapse	NM_024012.2:776
PLXNB3-mRNA	-0.717	0.157	-1.03	-0.409	0.608	0.491	0.753	0.000171	0.155	lm.nb	Neurogenesis	NM_001163257.1:3663
MAG-mRNA	-1.08	0.25	-1.57	-0.591	0.473	0.337	0.664	0.000301	0.228	lm.nb	Myelogenesis, Oligodendrocyte DifferentiationMaturation	NM_001199216.1:1780
DMD-mRNA	0.41	0.0988	0.216	0.604	1.33	1.16	1.52	0.000454	0.245	lm.nb	Blood Brain Barrier, Cytoskeletal Dynamics, Neurogenesis	NM_004014.2:1334
POR-mRNA	0.524	0.128	0.274	0.774	1.44	1.21	1.71	0.000501	0.245	lm.nb	NO Metabolism and Signaling	NM_000941.2:114
CTSB-mRNA	0.745	0.184	0.384	1.11	1.68	1.31	2.15	0.00058	0.245	lm.nb	Apoptosis, Autophagy, Microglia Neurodegenerative Phenotype (MGnD), Primed Microglia, Stage 1 DAM	NM_147780.2:1054
BHLHE40-mRNA	0.682	0.171	0.348	1.02	1.6	1.27	2.02	0.000647	0.245	lm.nb	Circadian Signaling, Microglia Neurodegenerative Phenotype (MGnD)	NM_003670.2:716
VEGFA-mRNA	1.18	0.294	0.599	1.75	2.26	1.52	3.37	0.000653	0.245	lm.nb	Angiogenesis, Hypoxia, Microglia Neurodegenerative Phenotype (MGnD), Neurogenesis	NM_001025366.1:1325
SPI1-mRNA	1.09	0.275	0.55	1.63	2.13	1.46	3.09	0.000711	0.245	lm.nb	Microglia Neurodegenerative Phenotype (MGnD)	NM_003120.1:730
SERPINA3-mRNA	1.64	0.417	0.826	2.46	3.12	1.77	5.5	0.00075	0.245	lm.nb	Astrocyte Markers	NM_001085.4:1220
APLP2-mRNA	0.668	0.172	0.33	1.01	1.59	1.26	2.01	0.000869	0.245	lm.nb	Primed Microglia	NM_001642.2:1380
NPAS2-mRNA	0.549	0.142	0.271	0.828	1.46	1.21	1.78	0.000895	0.245	lm.nb	Circadian Signaling	NM_002518.3:1458
ERMN-mRNA	-0.708	0.183	-1.07	-0.348	0.612	0.477	0.785	0.000908	0.245	lm.nb	Cytoskeletal Dynamics, Oligodendrocyte Markers	NM_001304344.1:440
HMOX1-mRNA	1.2	0.31	0.588	1.8	2.29	1.5	3.49	0.000921	0.245	lm.nb	Hypoxia	NM_002133.2:781
TPI1-mRNA	0.357	0.0944	0.172	0.542	1.28	1.13	1.46	0.00109	0.274	lm.nb	Glucose Metabolism, Primed Microglia	NM_000365.5:153
DRD1-mRNA	1.15	0.307	0.549	1.75	2.22	1.46	3.37	0.00118	0.281	lm.nb	Astrocyte DifferentiationFunction, Calcium Signaling, Dopaminergic Synapse, Neuroactive Ligands and Receptors	NM_000794.3:1970
TMEM88B-mRNA	-1.07	0.294	-1.64	-0.492	0.477	0.32	0.711	0.00156	0.341	lm.nb		NM_001146685.1:222
STAMBPL1-mRNA	0.922	0.254	0.424	1.42	1.89	1.34	2.68	0.00158	0.341	lm.nb	Primed Microglia	NM_020799.2:1600
PLP1-mRNA	-0.78	0.22	-1.21	-0.35	0.582	0.432	0.785	0.0019	0.378	lm.nb	Astrocyte DifferentiationFunction, Myelogenesis, Oligodendrocyte DifferentiationMaturation, Oligodendrocyte Markers	NM_000533.3:758
PGK1-mRNA	0.746	0.211	0.334	1.16	1.68	1.26	2.23	0.00192	0.378	lm.nb	Glucose Metabolism	NM_000291.2:1030
HSP90AB1-mRNA	0.697	0.199	0.307	1.09	1.62	1.24	2.13	0.00211	0.379	lm.nb	Cytoskeletal Dynamics, Inflammasome, Proteotoxic Stress	NM_007355.3:1530

**Table 5:** Top 25 differentially expressed genes in COVID vs. Non-COVID in MFG

	Log2 fold change	std error (log2)	Lower confidence limit (log2)	Upper confidence limit (log2)	Linear fold change	Lower confidence limit (linear)	Upper confidence limit (linear)	P-value	BY.p.value	method	Gene.sets	probe.ID
NDUFA10-mRNA	-0.285	0.0764	-0.435	-0.135	0.821	0.74	0.91	0.00123	1	lm.nb	Cannabinoid Signaling	NM_004544.3:876
SERPINA3-mRNA	2.22	0.649	0.953	3.5	4.67	1.94	11.3	0.00252	1	lm.nb	Astrocyte Markers	NM_001085.4:1220
CHI3L1-mRNA	2.06	0.619	0.848	3.27	4.17	1.8	9.68	0.00318	1	lm.nb	Astrocyte Markers	NM_001276.2:475
EGR1-mRNA	1.52	0.473	0.588	2.44	2.86	1.5	5.43	0.00426	1	lm.nb	Cytokines, Interferon Signaling, Microglia Neurodegenerative Phenotype (MGnD), Microglial Markers	NM_001964.2:838
EIF2AK1-mRNA	-0.444	0.141	-0.721	-0.167	0.735	0.607	0.891	0.00495	1	lm.nb	Proteotoxic Stress	NM_014413.2:1685
CTSF-mRNA	-0.259	0.0827	-0.421	-0.0967	0.836	0.747	0.935	0.00507	1	lm.nb	Apoptosis, Microglial Markers	NM_003793.3:655
GNAS-mRNA	-0.412	0.138	-0.682	-0.142	0.752	0.623	0.906	0.00697	1	lm.nb	Calcium Signaling, Dopaminergic Synapse, Glutamatergic Synapse, Microglia Neurodegenerative Phenotype (MGnD), Serotonergic Synapse	NM_080425.1:1910
ROCK2-mRNA	-0.229	0.0835	-0.393	-0.0652	0.853	0.762	0.956	0.0122	1	lm.nb	Cell Migration, Wnt Signaling	NM_004850.3:3140
HSPA1A-mRNA	1.5	0.556	0.406	2.58	2.82	1.32	6	0.0137	1	lm.nb	Microglial Markers, Proteotoxic Stress	NM_005345.5:1875
TBK1-mRNA	-0.341	0.127	-0.59	-0.0915	0.79	0.664	0.939	0.0141	1	lm.nb	Autophagy, Cytokines, NF-kappaB Signaling	NM_013254.2:1610
NLK-mRNA	-0.396	0.15	-0.69	-0.101	0.76	0.62	0.932	0.0156	1	lm.nb	Wnt Signaling	NM_016231.4:618
TIAM1-mRNA	-0.315	0.122	-0.554	-0.0767	0.804	0.681	0.948	0.0171	1	lm.nb	Cytoskeletal Dynamics, Neurogenesis, Neurotrophin Signaling	NM_001353691.1:4520
TRIM37-mRNA	-0.416	0.161	-0.732	-0.101	0.749	0.602	0.932	0.0171	1	lm.nb	Antigen Processing and Presentation	NM_015294.4:1910
CD44-mRNA	0.979	0.379	0.236	1.72	1.97	1.18	3.3	0.0174	1	lm.nb	Astrocyte Markers, Interferon Signaling, Primed Microglia	NM_001001392.1:429
VEGFA-mRNA	0.777	0.304	0.182	1.37	1.71	1.13	2.59	0.0183	1	lm.nb	Angiogenesis, Hypoxia, Microglia Neurodegenerative Phenotype (MGnD), Neurogenesis	NM_001025366.1:1325
ACADM-mRNA	-0.252	0.0995	-0.447	-0.0569	0.84	0.734	0.961	0.0194	1	lm.nb	Lipid Metabolism	NM_000016.5:1289
GSN-mRNA	-0.442	0.178	-0.791	-0.0932	0.736	0.578	0.937	0.0215	1	lm.nb	Cytoskeletal Dynamics, Oligodendrocyte DifferentiationMaturation, Phagocytosis	NM_000177.4:1075
P2RY11-mRNA	-0.628	0.257	-1.13	-0.124	0.647	0.456	0.918	0.0236	1	lm.nb	Neuroactive Ligands and Receptors, Purinergic Signaling	NM_002566.4:729
Figure 4-mRNA	-0.408	0.169	-0.74	-0.0772	0.753	0.599	0.948	0.0249	1	lm.nb	Myelogenesis, Neurogenesis	NM_014845.5:1060
KCTD12-mRNA	-0.527	0.228	-0.973	-0.0803	0.694	0.509	0.946	0.031	1	lm.nb	GABAergic Synapse	NM_138444.2:450
KCND1-mRNA	-0.571	0.247	-1.06	-0.0861	0.673	0.481	0.942	0.0313	1	lm.nb	Ion Transport	NM_004979.4:2400
USP2-mRNA	-0.32	0.139	-0.592	-0.0479	0.801	0.664	0.967	0.0315	1	lm.nb	Microglial Markers, Primed Microglia	NM_001243759.1:676
BECN1-mRNA	-0.311	0.135	-0.576	-0.0464	0.806	0.671	0.968	0.0315	1	lm.nb	Autophagy	NM_003766.2:810
LPL-mRNA	-0.499	0.218	-0.927	-0.0712	0.708	0.526	0.952	0.0327	1	lm.nb	Microglia Neurodegenerative Phenotype (MGnD), Stage 2 DAM	NM_000237.2:736

### Automated cell and protein coverage quantification

Quantification of IHC stains was performed using QuPath digital analysis [40] as previously performed and validated within our laboratory [31]. The positive cell detection tool was utilized for quantifying all glial stains and positive pixel count was used to quantify the percent of tissue covered by pathologies and PLP1.The scripts used for tissue annotation, pathological coverage quantification, and microglial cell count quantification are available in our laboratory’s previous manuscript [31], and were adjusted for the quantification of astrocytes and oligodendrocytes. Whole-tissue scanning at 20x magnification of fluorescent sections was performed using an Oympus VS200 (Olympus, Japan), with manually applied focus points. QuPath was also used for colocalization and proximity analysis of whole-tissue immunofluorescent scans. For colocalization measurements, the pixel classifier/thresholder tool was used to first create annotations for CD31 followed by the creation of GFAP annotations within these boundaries. The percentage overlap for the areas of these two annotations was determined in Excel using the measurement outputs from QuPath. For vessel proximity analysis, a base script provided by image.sc user Egor Zindy (EP.Zindy) [41] was modified to automate the creation of 7 concentric rings, each with a radius of 10 μm, around CD31-positive vessels across an entire brain region (i.e. the hippocampus). Within areas on the tissue where there are less than 70 μm between adjacent blood vessels, ring annotations will merge as to accurately quantify the distance from the nearest blood vessel while taking into account the surrounding ones as well. QuPath cell detection plugin StarDist [42] was then used to quantify GFAP-positive astrocytes within each of these rings to obtain proximity-based densities of the cell type.

### Frozen tissue RNA isolation and quality analysis

Human mRNA was isolated from fresh frozen frontal cortex tissue that was kept at -80 °C for long-term storage. One 25–35 mg chunk of tissue was disrupted into a fine powder in a pre-chilled mortar and pestle with liquid nitrogen added to the mortar to prevent thawing of the tissue. The powdered tissue was then transferred to pre-chilled, RNAse-free tubes and 500 µL of TRIzol reagent (ThermoFisher Scientific, USA) was added. The TRIzol Reagent user guide provided by ThermoFisher Scientific (https://assets.thermofisher.com/TFS-Assets/LSG/manuals/trizol_reagent.pdf) was then followed until step 7 (“Tissues” starting material, volumes adapted for 500 µL starting TRIzol). The aqueous phase from step 7 was then purified using columns from the Qiagen RNeasy Mini Kit (Cat. Number 74104) beginning from step 5 of the protocol. Isolated RNA was eluted twice (first by loading water onto the column and then by reloading the eluate onto the column) into RNAse-free tubes and stored at -20 °C for short-term use and − 80 °C for archival storage. Quality analysis was performed on the Bioanalyzer 2100 (Agilent, Denmark) platform, through which the RNA integrity number (RIN) and DV200 were calculated. Since we only planned to use the isolated RNA for high cycle count RT-qPCR detection of COVID transcripts and not for other protocols requiring higher sensitivity (i.e. RNA-seq or accurate quantification of low-expressed genes), we settled on a RIN cut-off of 3.0 for analyzed cases. RNA concentration was determined using a Qubit 4 fluorometer (Invitrogen, USA), and stock solutions were diluted to 100 ng/µL.

### RT-qPCR detection of COVID-19 mRNA

RT-qPCR detection of COVID-19 transcripts was performed using the Promega GoTaq Probe 1-Step RT-qPCR kit (Promega, USA) on a 384 well-plate. 5 µL reactions were set up with 2.5 µL GoTaq probe master mix, 0.1 µL GoScript RT mix, 0.2 each of premixed primer/probe for N1 and N2 (for viral detection reactions) or 0.2 each of premixed primer/probe for RNAseP and nuclease-free water (for reactions to determine RNA template viability) (Integrated DNA Technologies, USA catalog #10006713), and 2 µL of isolated RNA per well. Reactions were run in duplicate with positive controls for SARS-CoV-2 N and E RNA (Promega, USA catalog #AM2050) and negative control wells with nuclease-free water in place of RNA template. RT-qPCR was performed using the CFX384 Touch Real-Time PCR Detection System (BioRad, USA) with the cycling parameters included with the Promega GoTaq Probe 1-Step RT-qPCR kit modified to run for 45 cycles of amplification. Detection of viral RNA was defined as if the fluorescence for the N1 + N2 reactions for a case exceeded the threshold line and if the case generates a quantification cycle (Cq) value.

### FFPE tissue DNA isolation and qPCR genotyping for APOE

To determine patients’ ApoE genotypes, gDNA was extracted from a 10 μm scroll of FFPE temporal brain tissue using the QIAamp DNA FFPE Advanced Kit (Qiagen, Germany) following manufacturer-provided protocols. 35 µL of purified gDNA was eluted from the column, concentration was determined using a Qubit 4 fluorometer (Invitrogen, USA), and the samples were diluted to a concentration of 5 ng/µL. Cases with stock concentrations of gDNA lower than 5 ng/µL could not be confidently genotyped and were excluded from analysis. qPCR genotyping was performed in a 384-well plate using the CFX384 Touch Real-Time PCR Detection System (BioRad, USA). 5 µL reaction wells were set up with 2.5 µL Taqman Genotyping Master Mix (Fisher, USA catalog #4371355), 0.125 µL of Taqman SNP Genotyping Assay for either rs7412 (Fisher, USA catalog #4351376) or rs429358 (Fisher, USA catalog #4351376), 0.375 µL of nuclease-free water, and 2 µL of 5 ng/µL gDNA. The plate was sealed using a microseal ‘B’ seal (BioRad, USA catalog #MSB1001) and cycled 40 times with the following parameters: 10 min AmpliTaq Gold, UP Enzyme Activation @ 95 °C (1x); 15 s of denaturing @ 95 °C and 1 min of annealing/extension @ 60 °C (40x). Nucleotide identity at each SNP and genotype was determined using previously defined ratios between VIC: FAM fluorescence.

### FFPE tissue RNA isolation and quality analysis

RNA was isolated from all COVID and Non-COVID patients within the cohort (*N* = 23) using the High Pure FFPE RNA Isolation Kit (Roche Life Science, Germany). Two 20 μm thick scrolls of FFPE tissue were collected from the hippocampus and MFG of each patient and placed into cassettes. RNA was extracted from the scrolls following the manufacturer-provided protocols [43]. The concentration of extracted RNA was quantified using the Qubit 4 fluorometer (Invitrogen, USA), and RNA quality (DV200) was assessed via Bioanalyzer 2100 (Agilent, Denmark). Samples were stored in RNAse-free tubes at -20 °C overnight prior to running the nCounter^®^ assays, and were stored at -80 °C for long-term archival storage.

### NanoString nCounter^®^ gene expression analysis

Stock FFPE RNA was diluted with non-DEPC-treated nuclease-free water (Invitrogen, USA) using the previously collected Qubit and DV200 values to 20 ng/mL of mRNA with length ≥ 200 nucleotides as described by NanoString [44]. 5 µL of each diluted sample was hybridized with the NanoString nCounter^®^ Glial Profiling Panel (XT HS Glial Profiling #115000429) in a thermocycler for 18 h at 65 °C (heated lid at 72 °C) following NanoString’s provided protocols [31, 45]. Detection using the NanoString nCounter^®^ Profiler and data analysis and quality assessment via nSolver^®^ was performed exactly as previously described [31].

### Statistics

Statistical analysis was performed on GraphPad Prism 9 and individual parameters are reported in the figure captions.

## Results

### Cohort overview and regional differences in COVID-AD

To identify the effect of COVID-19 infection on regions affected by AD pathology at varying points of disease progression, four regions, including the medial frontal gyrus (MFG), superior temporal gyrus (STG), hippocampus, and combined fusiform and inferior temporal gyri (FG + ITG), were sampled for immunohistochemical analysis (Fig. 1A). As previous literature has heavily implicated the choroid plexus and blood-CSF barrier in the transmission of peripheral COVID-19-associated inflammatory signals to the brain [14], the selected regions were also arranged in terms of anatomical proximity to the lateral ventricles [46–48] (Fig. 1A), with the hippocampus being the closest and the MFG the furthest. The grey matter (GM) of the hippocampus was further classified into the four cornu ammonis (CA) subfields (CA1-CA4), subiculum (SUB), and entorhinal cortex (EC) (Fig. 1B) and the STG and MFG into cortex (grey natter) and white matter (WM) regions (Fig. 1C). FG + ITG regions were attached to hippocampal sections when collected at autopsy but were separated during QuPath annotation and considered as their own independent regions during quantification (Fig. 1B). These regions were assessed in patients with high ADNC who were separated into groups based on if they suffered and recovered from at least one COVID-19 infection (COVID), did not report a COVID-19 infection and died during the same time period as COVID patients (Non-COVID), and AD patients who died prior to SARS-CoV-2 arrival in the US (Pre-2020) (see methods for additional selection criteria) (Fig. 1D). Additionally, patients in all three cohorts were negative for SARS-CoV-2 mRNA via RT-qPCR detection, indicating that the observed changes below persist in the parenchyma following the resolution of active infection.

### COVID-AD survivors show elevated Aβ plaque burden in select brain regions

**Fig. 2:**
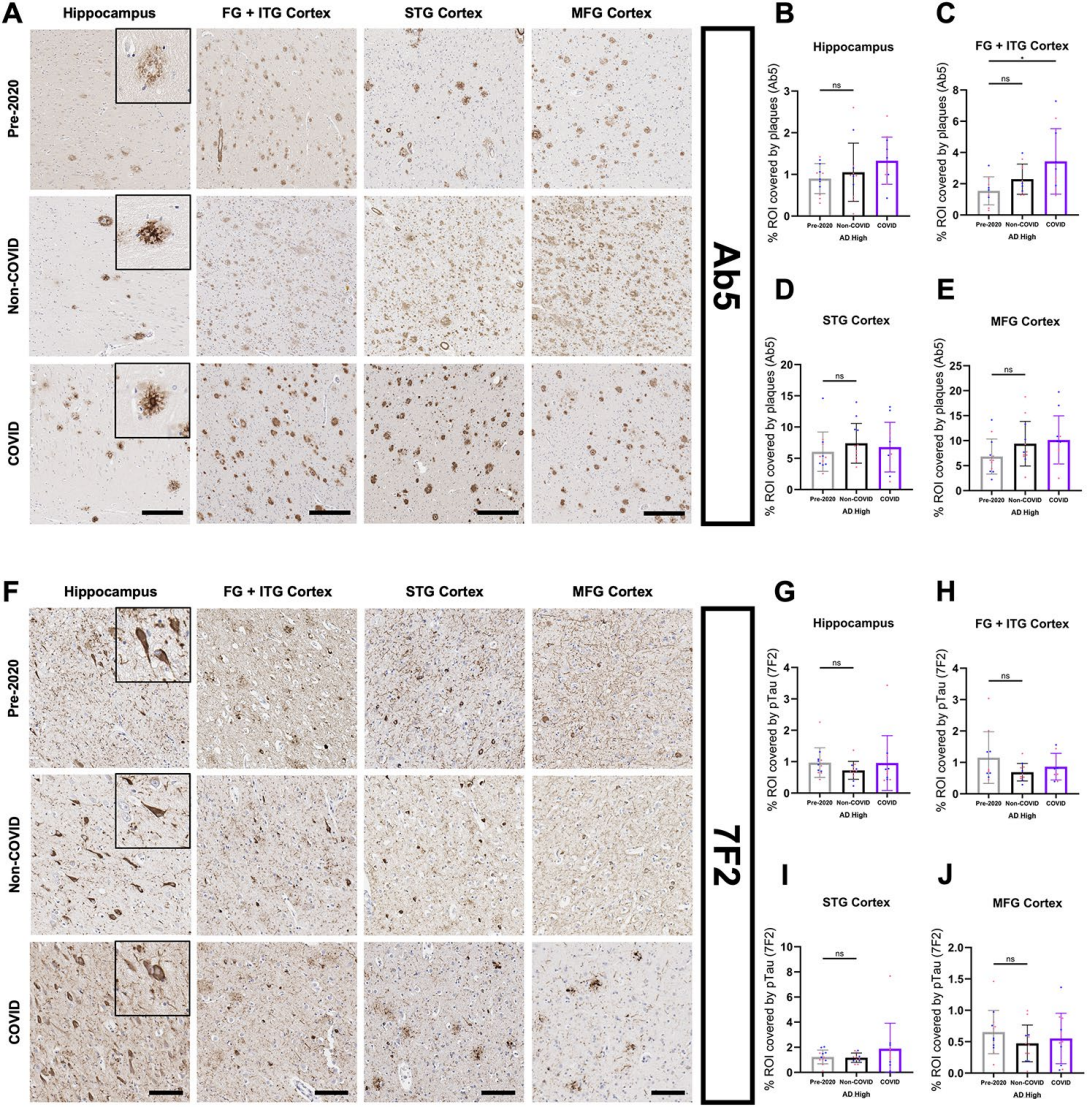
COVID-AD survivors show elevated Aβ plaque burden in select brain regions. (**A**) Representative images of Ab5 (Aβ plaques) immunopositivity in the hippocampus grey matter, FG + ITG cortex, STG cortex, and MFG cortex of cohort patients. Inserts depict morphology of pathological protein deposits in each patient cohort. Tissue coverage was quantified in 5 μm-thick FFPE sections as described in the methods. Scale bar = 300 μm. Percent coverage of cortex (grey matter in the case of hippocampus) by Ab5 in the (**B**) hippocampus (Pre-2020 *N* = 12, Non-COVID *N* = 12, and COVID *N* = 11), (**C**) FG + ITG (Pre-2020 *N* = 9, Non-COVID *N* = 11, and COVID *N* = 10), (**D**) STG (Pre-2020 *N* = 12, Non-COVID *N* = 12, and COVID *N* = 11), and (**E**) MFG (Pre-2020 *N* = 12, Non-COVID *N* = 12, and COVID *N* = 11) of the cohort patients. (**F**) Representative images of 7F2 (NFT) immunopositivity in the same regions and patients. Scale bar = 100 μm. **(G-J)** Percent coverage of the cortex in cohort patients by 7F2 in the (**G**) hippocampus (Pre-2020 *N* = 12, Non-COVID *N* = 12, and COVID *N* = 11), **(H)** FG + ITG (Pre-2020 *N* = 10, Non-COVID *N* = 11, and COVID *N* = 9), **(I)** STG (Pre-2020 *N* = 12, Non-COVID *N* = 12, and COVID *N* = 11), and **(J)** MFG (Pre-2020 *N* = 12, Non-COVID *N* = 12, and COVID *N* = 11). Comparisons were performed using an ordinary one-way ANOVA with Tukey’s multiple comparisons test, with a single pooled variance. Each group’s standard deviation is represented by error bars. “ns” = no significance, *p < 0.05, **p < 0.01, ***p < 0.001. Any comparisons lacking asterisks have no significance. The hippocampus regions contains the average of each patients’ subfield quantifications (Figs. 3C and F)

**Fig. 3:**
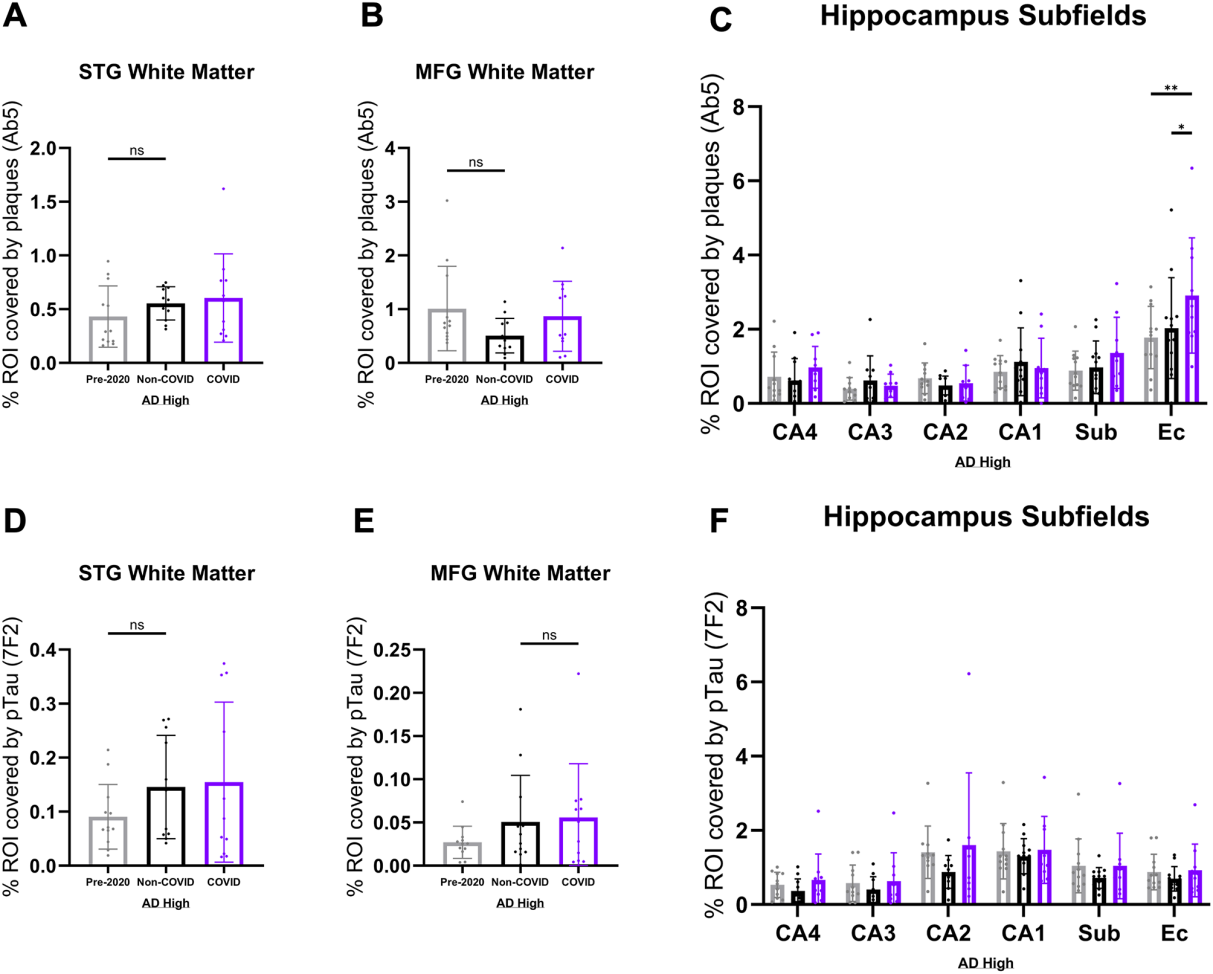
Quantification of white matter and hippocampal subfield AD pathology burden. QuPath quantification of plaque burden (Ab5) (**A-B**) and pTau burden (7F2) (**D-E**) in the STG and MFG white matter of cohort patients (Pre-2020 *N* = 12, Non-COVID *N* = 12, and COVID *N* = 11). Comparisons were performed using an ordinary one-way ANOVA with Tukey’s multiple comparisons test, with a single pooled variance. Hippocampal subfields were separated by annotation and analyzed in a similar manner for Ab5 coverage (**C**) and 7F2 coverage (**F**). Subfield comparisons were made using an ordinary two-way ANOVA with Tukey’s multiple comparisons test, with a single pooled variance. “ns” = no significance, *p < 0.05, **p < 0.01, ***p < 0.001. Any comparisons lacking asterisks are non-significant

To test if COVID-19 infection impacted AD-related protein pathologies, we quantified the burden of Aβ plaques (anti-Ab5 [49]) (Fig. 2A) and tau/NFT (anti-7F2 [50]) (Fig. 2F) in COVID, Non-COVID and Pre-2020 patients. Intriguingly, COVID patients showed higher Aβ coverage in the FG + ITG cortices (Fig. 2C) and EC (Fig. 3C) compared to Non-COVID and Pre-2020 patients. A similar trend toward elevated Aβ burden was also observed in the other brain regions evaluated (Fig. 2B, D-E). NFT coverage was largely unchanged between patient groups across all regions and hippocampal subfields (Figs. 2G-J and 3F).

### COVID-AD is characterized by elevated numbers of hippocampal microglia

**Fig. 4:**
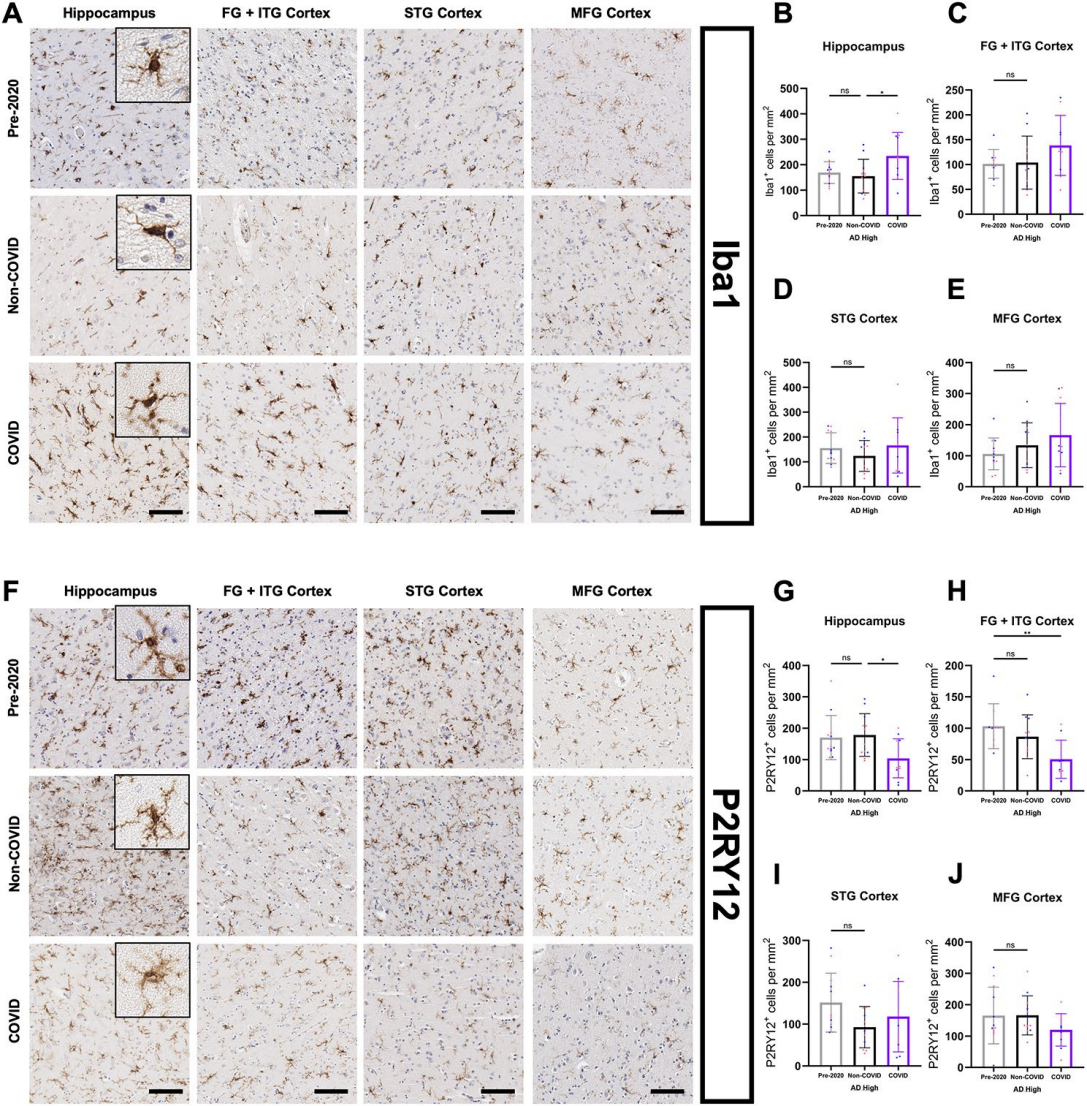
COVID-AD is characterized by elevated numbers of hippocampal microglia and loss of homeostatic microglia. (**A**) Images of Iba1-positive microglia in the hippocampus, FG + ITG, STG, and MFG of Pre-2020, Non-COVID, and COVID patients. Inserts depict cellular morphology in each patient cohort. Iba1-positive microglia appeared morphologically diverse, ranging from moderately ramified to amoeboid in terms of process extension. Quantification of Iba1-positive microglia per mm^2^ in the cortex/grey matter of (**B**) hippocampus (Pre-2020 *N* = 12, Non-COVID *N* = 12, and COVID *N* = 11), (**C**) FG + ITG (Pre-2020 *N* = 9, Non-COVID *N* = 11, and COVID *N* = 10), (**D**) STG (Pre-2020 *N* = 12, Non-COVID *N* = 12, and COVID *N* = 11), and (**E**) MFG (Pre-2020 *N* = 12, Non-COVID *N* = 12, and COVID *N* = 11). Hippocampal values were obtained by averaging the Iba1 quantifications in the subfields of each patient as reported below (Fig. 5C). (**F**) Panel depicting P2RY12-positive microglia distribution in the regions and patients mentioned above. Morphologically, P2RY12-positive microglia were highly ramified and possessed long-reaching processes. Quantification of P2RY12-positive microglia per mm^2^ in the cortex/grey matter of (**G**) hippocampus (Pre-2020 *N* = 12, Non-COVID *N* = 12, and COVID *N* = 11), **(H)** FG + ITG (Pre-2020 *N* = 9, Non-COVID *N* = 11, and COVID *N* = 10), **(I)** STG (Pre-2020 *N* = 12, Non-COVID *N* = 12, and COVID *N* = 11), and **(J)** MFG (Pre-2020 *N* = 12, Non-COVID *N* = 12, and COVID *N* = 11) in each experimental group. Subfield quantification data for the hippocampus region is provided below (Fig. 5F). IHC was performed in 10 μm-thick FFPE sections for both Iba1 and P2RY12. Comparisons were performed using an ordinary one-way ANOVA with Tukey’s multiple comparisons test, with a single pooled variance. Each group’s standard deviation is represented by error bars. “ns” = no significance, *p < 0.05, **p < 0.01, ***p < 0.001. Any comparisons lacking asterisks have no significance. Scale bars = 100 μm

To get an initial picture of microglia in COVID patients, we performed IHC analysis of ionized calcium binding adaptor molecule 1 (Iba1) (Fig. 4A) which is a cytoplasmic protein that acts as a pan-microglial marker in humans [51]. Using automated cell counting via the QuPath software [31] (see methods section), an increase in Iba1-positive microglia was observed in the hippocampi of COVID compared to Non-COVID (Fig. 4B), with the latter not differing significantly from Pre-2020 patients, validating the Non-COVID group as being representative of a non-infected population with respect to microglia numbers.

**Fig. 5:**
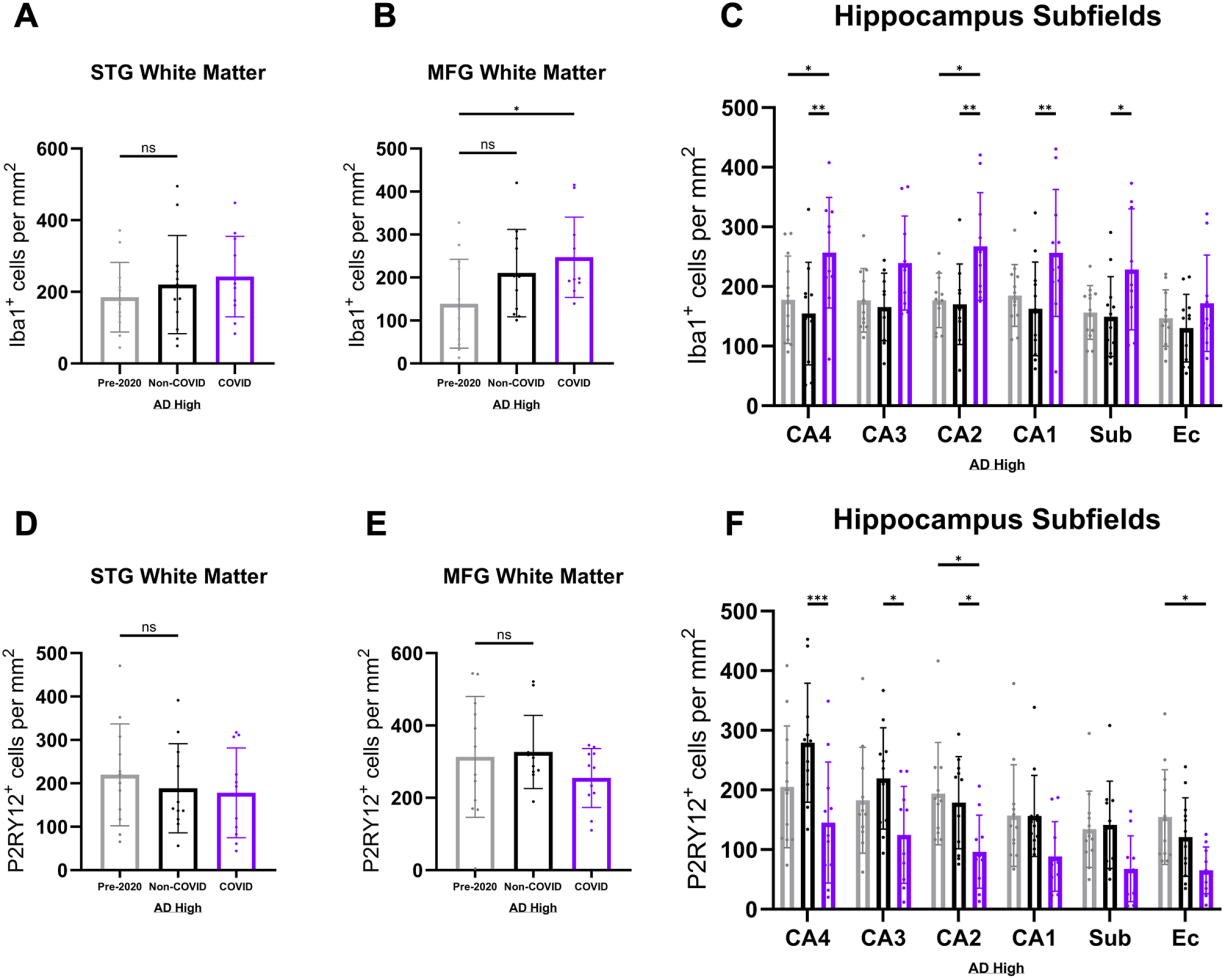
White matter and hippocampal subfield analysis of total and homeostatic microglia in COVID-AD. Iba1-positive (**A-B**) and homeostatic P2RY12-positive (**D-E**) microglia per mm^2^ in the STG and MFG white matter of Pre-2020 (*N* = 12), Non-COVID (*N* = 12), and COVID (*N* = 11) patients. Comparisons were made using an ordinary one-way ANOVA with Tukey’s multiple comparisons test, with a single pooled variance. Hippocampal subfield quantification of Iba1-positive (**C**) and P2RY12-positive (**F**) microglia per mm^2^ was also performed to determine spatial location of profile changes in the hippocampus. Subfield comparisons were made using an ordinary two-way ANOVA with Tukey’s multiple comparisons test, with a single pooled variance. Each group’s standard deviation is represented by error bars. “ns” = no significance, *p < 0.05, **p < 0.01, ***p < 0.001. Any comparisons lacking asterisks are non-significant

These changes were most apparent in the CA4, CA2, CA1 subfields and the subiculum of COVID AD (Fig. 5C). While other regions further away from the CP exhibit similar trends towards higher microglia numbers in COVID (Fig. 4C-E), these only reached statistical significance in the MFG WM (Fig. 5B), suggesting that anatomically “deeper” regions closer to the choroid plexus could be affected more acutely by a systemic infectious stimulus. Additionally, the number of Iba1-positive microglia was not significantly associated with Aβ plaque burden (Supp. Figure 1 A), confirming that our findings were not primarily driven by patient-to-patient variances in pathology.

### Compounding loss of microglial homeostasis in COVID-AD patients

To further characterize the activation state of microglial populations in the COVID group, we evaluated purinergic receptor P2Y12 (P2RY12) (Fig. 4F), a transmembrane protein expressed by highly ramified microglia that has previously been shown to decrease in expression with increasing microglial activation [52, 53]. Individuals suffering from AD have been shown to have reduced numbers of homeostatic, P2RY12-positive microglia [18, 20] as a response to disease -specific protein pathology. In line with our Iba1-data, COVID patients showed reduced numbers of P2RY12-positive microglia in the hippocampus (Fig. 4G) and the FG/ITG cortex (Fig. 4H) as compared to the Non-COVID and Pre-2020 patients. This loss of microglial homeostasis was mainly detected in the CP-adjacent CA4-CA2 regions but could also be detected in the EC (Fig. 5F). STG and MFG cortices did not significantly differ between groups in terms of P2RY12-positive microglia (Fig. 4I-J).

**Fig. 6:**
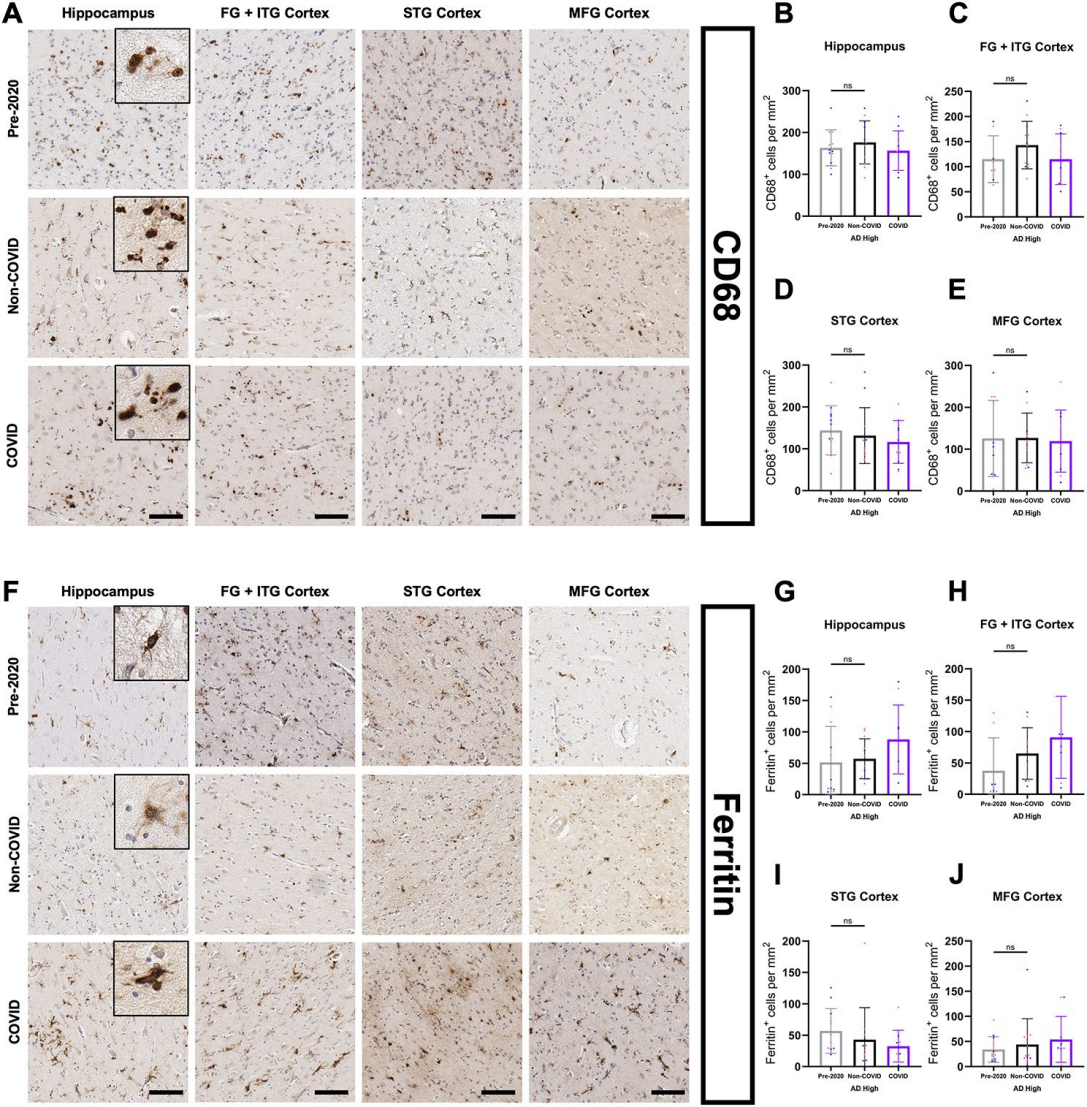
Assessment of microglial activation profiles in COVID-AD patients. (**A**) Representative images of CD68-positive microglia in hippocampus grey matter, FG + ITG cortex, STG cortex, and MFG cortex in our three patient groups. Inserts depict cellular morphology for each marker. CD68-positive microglia morphology was largely spherical and ameboid, with a distinct loss of process length/extension. QuPath quantification of CD68-positive microglia per mm^2^ in (**B**) hippocampus grey matter (Pre-2020 *N* = 12, Non-COVID *N* = 12, and COVID *N* = 11), (**C**) FG + ITG cortex (Pre-2020 *N* = 8, Non-COVID *N* = 12, and COVID *N* = 10), (**D**) STG cortex (Pre-2020 *N* = 12, Non-COVID *N* = 12, and COVID *N* = 11), and (**E**) MFG cortex (Pre-2020 *N* = 12, Non-COVID *N* = 12, and COVID *N* = 11) in cohort patients. Measurements for the hippocampus come from the average of subfields CA4-EC (Fig. 7C). (**F**) Panel depicting ferritin-positive microglia in the hippocampal grey matter, FG + ITG cortex, STG cortex, and MFG cortex of our three patient groups. Ferritin-positive microglia appeared morphologically dystrophic, with retracted and “shriveled” processes. QuPath quantification of ferritin-positive microglia per mm^2^ in (**G**) hippocampus grey matter (Pre-2020 *N* = 12, Non-COVID *N* = 12, and COVID *N* = 11), (**H**) FG + ITG cortex (Pre-2020 *N* = 8, Non-COVID *N* = 12, and COVID *N* = 10), (**I**) STG cortex (Pre-2020 *N* = 12, Non-COVID *N* = 12, and COVID *N* = 11), and (**J**) MFG cortex (Pre-2020 *N* = 12, Non-COVID *N* = 12, and COVID *N* = 11). Hippocampus means were derived from subfield averages (Fig. 7F). IHC was performed was performed in 10 μm-thick FFPE sections for both markers. Comparisons were performed using an ordinary one-way ANOVA with Tukey’s multiple comparisons test, with a single pooled variance. Each group’s standard deviation is represented by error bars. “ns” = no significance, *p < 0.05, **p < 0.01, ***p < 0.001. Any comparisons lacking asterisks have no significance. Scale bars = 100 μm

To study additional markers of microglial activation, we next analyzed cluster of differentiation 68 (CD68) – a lysosomal transmembrane protein and a marker for phagocytic activity in microglia [54, 55] (Fig. 6A). This analysis did not exhibit significant differences in numbers of positive cells between COVID and Non-COVID groups irrespective of brain region (Fig. 6B-E), suggesting that there is no major association between COVID infection and phagocytic activity of microglia in AD patients. Next, we studied ferritin, a primarily cytosolic protein complex composed of light and heavy (FTH1) chains enabling iron uptake and storage [56] (Fig. 6F). Previous studies have demonstrated an association between microglial ferritin expression and microglial dystrophy in patients with AD [57–59].

**Fig. 7:**
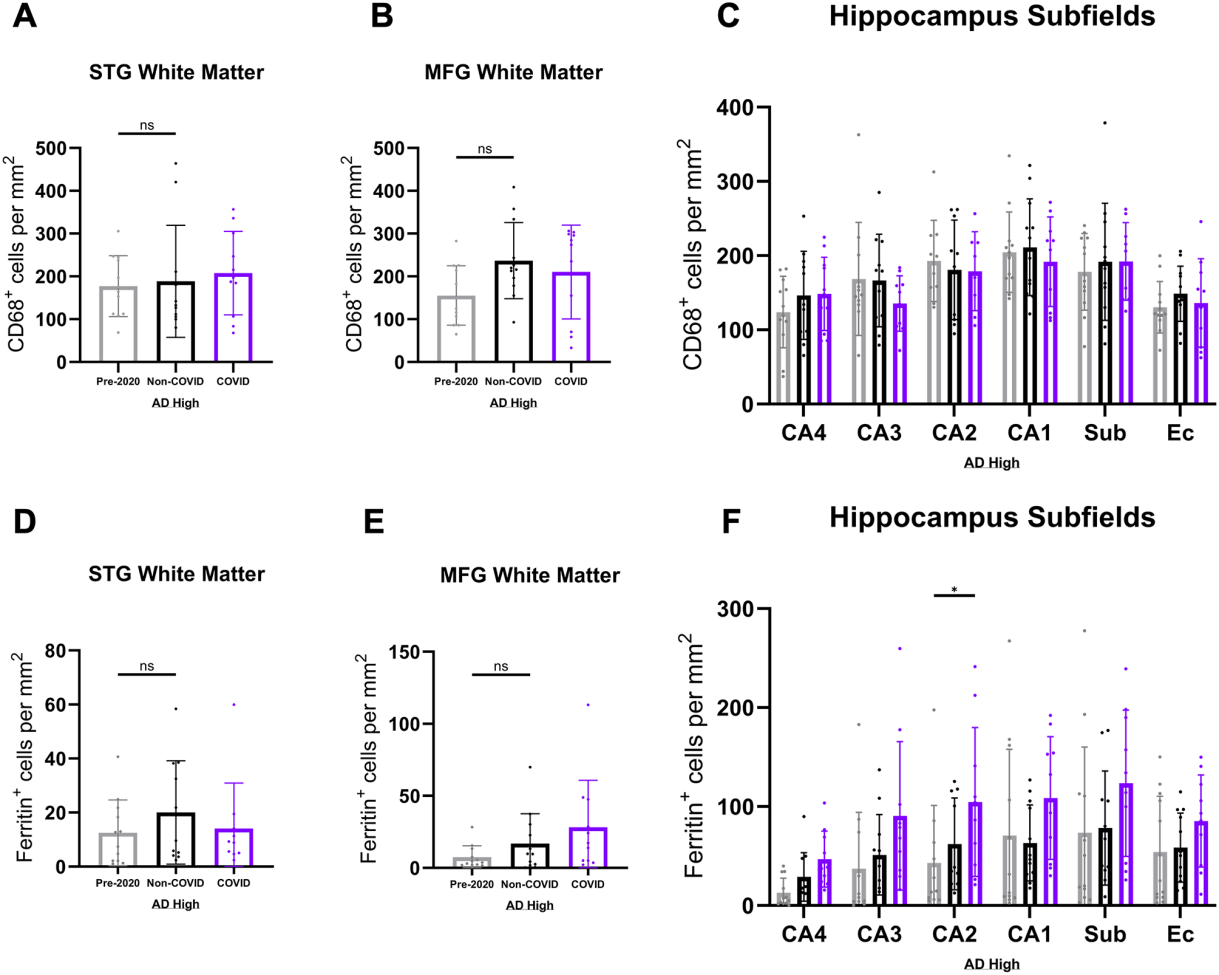
White matter and hippocampal subfield microglial activation profiles. Measurements for CD68-positive (**A-B**) and Ferritin-positive (**D-E**) microglia per mm^2^ in the STG and MFG white matter of Pre-2020 (*N* = 12), Non-COVID (*N* = 12), and COVID (*N* = 11) patients. Comparisons were made using an ordinary one-way ANOVA with Tukey’s multiple comparisons test, with a single pooled variance. We also performed hippocampal subfield quantification of CD68-positive (**C**) and Ferritin-positive (**F**) microglia per mm^2^. Subfield comparisons were made using an ordinary two-way ANOVA with Tukey’s multiple comparisons test, with a single pooled variance. Each group’s standard deviation is represented by error bars. “ns” = no significance, *p < 0.05, **p < 0.01, ***p < 0.001. Any comparisons lacking asterisks are non-significant

Analyzing ferritin-positive microglia, we noticed a trend towards increased microglia dystrophy in the hippocampus, FG + ITG cortices, and MFG cortex of COVID compared to the Non-COVID and Pre-2020 groups (Fig. 6G-J), with significantly higher numbers of Ferritin positive cells observed in the CA2 subfield (Fig. 7F). The number of positive cells for each of these three markers was again compared against Aβ burden (Supp. Figure 1E-P), revealing no significant associations between either marker and pathological burden.

### Astrocyte populations are universally diminished in COVID-AD

**Fig. 8:**
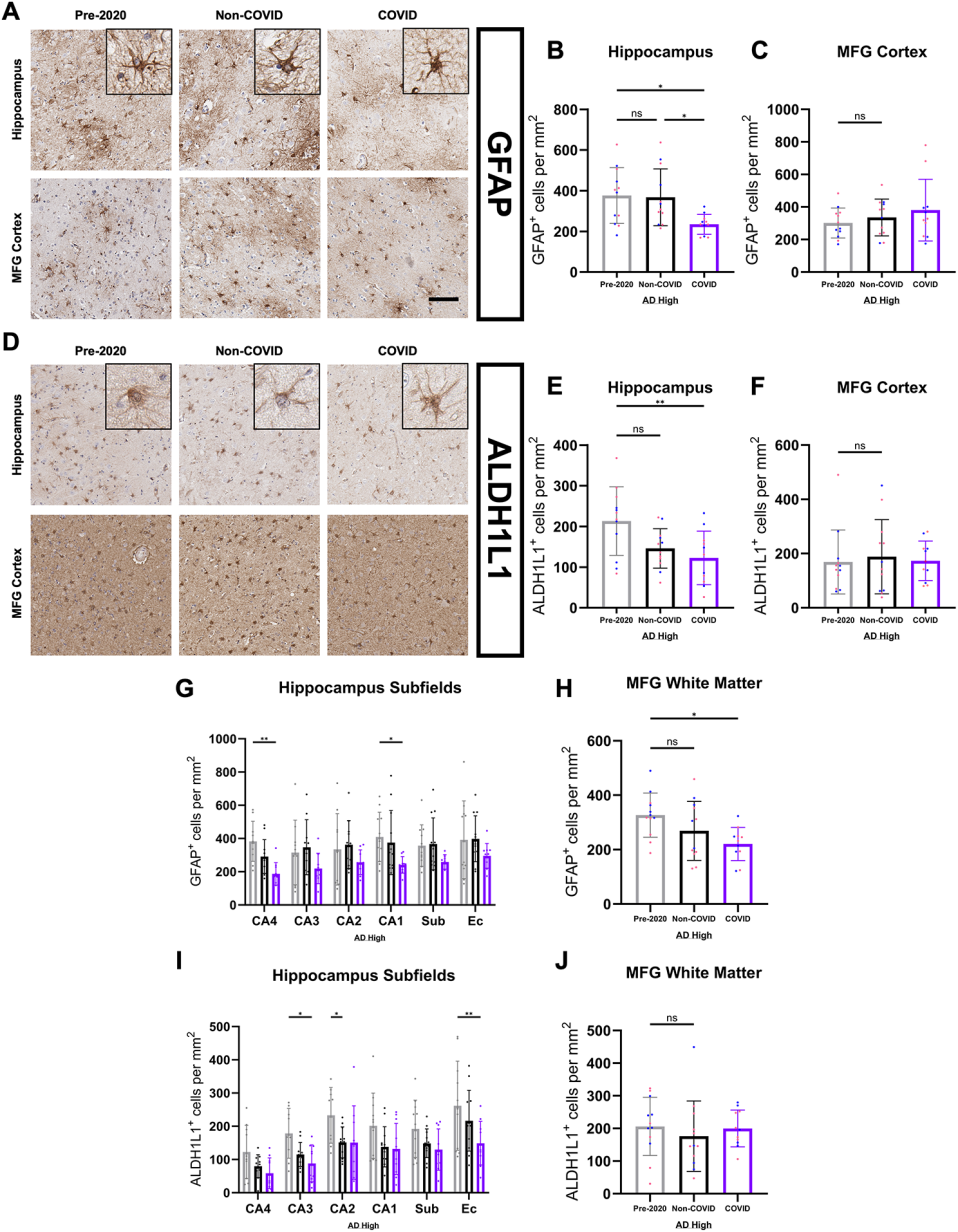
Astrocyte populations are universally diminished in COVID-AD. (**A**) Appearance of GFAP-positive astrocytes in the hippocampus and MFG of Pre-2020, Non-COVID, and COVID patients. GFAP-positive astrocytes displayed extended processes with filamentous patches in the vicinity of most cells. (**B**) QuPath quantification of hippocampal GFAP-positive cells revealed a significant reduction of reactive astrocyte number in COVID patients (Pre-2020 *N* = 12, Non-COVID *N* = 12, and COVID *N* = 11). Hippocampal measurements were calculated from an average of CA4-EC subfield measurements (Fig. 8G). (**C**) There was no observed difference in GFAP positivity across groups in the MFG cortex. (**D**) Quantification of ALDH1L1-positive astrocytes was performed in the hippocampi and MFG of our cohort groups to assess the extent to which the pan-astrocyte lineage is affected in COVID patients. ALDH1L1-positive astrocytes displayed similar morphology to their GFAP counterparts but lacked the previously observed filamentous patches. (**E**) QuPath quantification of ALDH1L1-positive astrocytes in the hippocampus revealed a significant reduction in number of the cell type in the COVID group (Pre-2020 *N* = 12, Non-COVID *N* = 12, and COVID *N* = 11). (**F**) Quantification of ALDH1L1-positive astrocytes in the MFG cortex revealed no differences across groups. Group averages were derived from subfield measurements (Fig. 8I). IHC was performed for both markers in 5 μm-thick FFPE sections. (**G-I**) Hippocampal subfield and (**H-J**) MFG white matter quantification of GFAP-positive and ALDH1L1-positive astrocytes. Comparisons were performed using an ordinary one-way ANOVA with Tukey’s multiple comparisons test, with a single pooled variance or an unpaired student’s t-test. Each group’s standard deviation is represented by error bars. “ns” = no significance, *p < 0.05, **p < 0.01, ***p < 0.001. Any comparisons lacking asterisks are non-significant. Scale bars = 100 μm

Interested in revealing a more comprehensive image of the neuroimmune status possessed by COVID patients, we performed IHC analysis of various astrocyte-lineage markers in the hippocampus and MFG. By analyzing astrocytes from two regions at opposite distances from the lateral ventricles, we sought to determine whether proximity to the CP was similarly associated with more acute differences between groups as observed within the microglial phenotypes. We first compared glial fibrillary acidic protein (GFAP) between groups (Fig. 8A), a marker for a subpopulation of reactive astrocytes that has been observed to proliferate in relation to Aβ pathological burden [26, 60–62]. Interestingly, COVID patients had significantly fewer numbers of hippocampal GFAP-positive astrocytes than both Non-COVID and Pre-2020 patients (Fig. 8B), with the most acute differences occurring in the CA4 and CA1 hippocampal subfields (Fig. 8G). While no difference in GFAP-positive astrocyte quantity was observed in the MFG cortex (Fig. 8C), COVID patients possessed significantly lower numbers of GFAP-positive astrocytes in the more CP-proximal MFG white matter (Fig. 8H).

In order to determine whether the reduction in astrocyte number was specific to the reactive sub-population or if astrocytes were universally affected in COVID patients, we decided to evaluate aldehyde dehydrogenase 1 family member L1 (ALDH1L1), a pan-astrocytic marker [63–67] (Fig. 8D). Concordant with our GFAP data, COVID patients had the least number of hippocampal ALDH1L1-positive cells out of the three groups (Fig. 8E), with significantly lower numbers as compared to the Pre-2020 cohort at the whole-region level. This astrocytic loss was most evident in the CA3 and EC subfields, and a difference between Pre-2020 and Non-COVID was observed in CA2 (Fig. 8I). No difference in MFG cortex (Fig. 8F) and white matter (Fig. 8J) ALDH1L1-positive astrocytes was observed. GFAP and ALDH1L1 were not significantly associated with plaque burden (Supp. Figure 1Q-R), suggestive of it not being a primary driving force for the observed astrocytic changes.

**Fig. 9:**
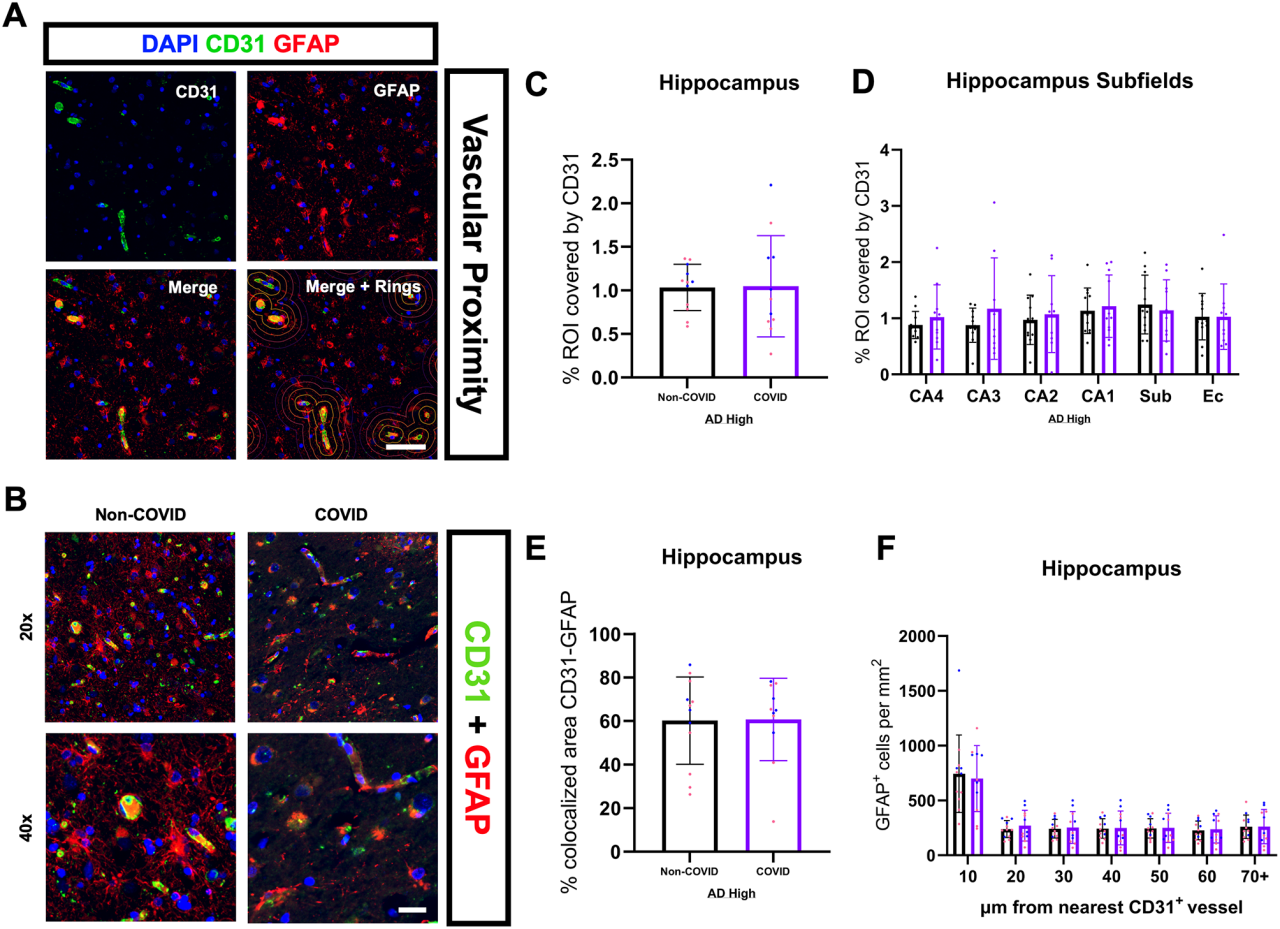
Proximity analysis of vasculature and astrocyte changes in COVID-AD. (**A**) Whole-tissue fluorescent scans of the hippocampus were taken using the Olympus VS200 platform at 20x magnification. Scans were loaded into QuPath, and a script was run to generate 7 concentric rings, each with a radius of 10 μm, around all CD31-positive blood vessels. The 10 μm radius was chosen to be able to completely fit an astrocyte soma within the ring. (**B**) Representative 20x and 40x images of fluorescent double-staining for CD31 and GFAP (Pre-2020 *N* = 12, Non-COVID *N* = 12, and COVID *N* = 11). (**C**) % ROI covered by CD31-positive vasculature, (**D**) subfield coverage by CD31, **(E)** GFAP-CD31 colocalization, and astrocyte-vessel proximity analysis were all performed in the same section. Comparisons were performed using an ordinary one-way ANOVA with Tukey’s multiple comparisons test, with a single pooled variance or an unpaired student’s t-test. Each group’s standard deviation is represented by error bars. “ns” = no significance, *p < 0.05, **p < 0.01, ***p < 0.001. Any comparisons lacking asterisks are non-significant. Brightfield scale bars = 100 μm. Fluorescent scale bars = 60 μm and 20 μm, respectively

Interested in further defining whether the observed astrocytic loss in COVID patients was the consequence of whole-region vulnerability or more localized changes at the BBB and parenchymal microvasculature, we performed immunofluorescent proximity analysis of platelet endothelial cell adhesion molecule (CD31 or PECAM-1)-positive endothelial cells and astrocytes (Fig. 9A-B). Pre-2020 patients were excluded from this analysis due to the presence of auto fluorescent artifacts in multiple cases within the cohort. CD31 is a transmembrane protein that acts as an endothelial cell marker throughout the body, labeling blood vessels within the brain parenchyma [68]. We decided to use GFAP as our marker for astrocytes due to its better morphological labeling of astrocyte processes and endfeet in combination with its more acute changes in the COVID cohort. Our analysis revealed no significant difference in hippocampal CD31-positive vessel coverage (Fig. 9C) and colocalization of CD31 and GFAP (i.e. astrocyte endfoot coverage) (Fig. 9E) between COVID and Non-COVID patients. Additionally, there was no observed loss of GFAP-positive astrocytes in proximity to cortical vasculature (Fig. 9F), suggestive of an alternate, hippocampus-specific factor not primarily associated with the BBB resulting in the loss of astrocytes observed in COVID patients.

### COVID-19 infection is associated with region-specific dysregulation of oligodendrocyte and myelin-associated pathways in AD

**Fig. 10:**
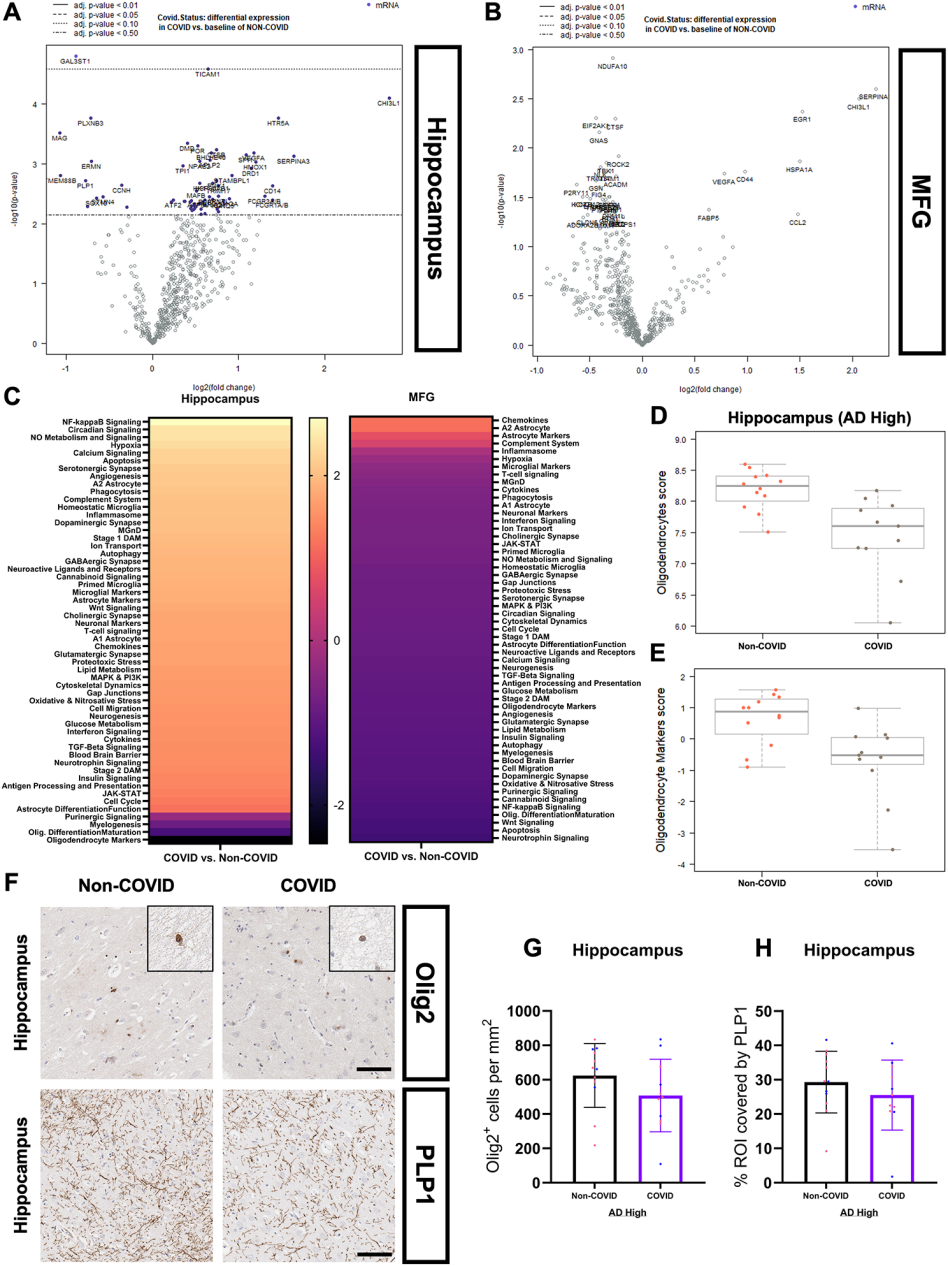
COVID-19 infection is associated with region-specific dysregulation of oligodendrocyte and myelin-associated pathways in AD. Volcano plots depicting DEGs in the hippocampus (**A**) and MFG (B) of COVID patients (*N* = 11) against a baseline of Non-COVID patients (*N* = 12) using the XT HS Glial Profiling panel on the NanoString nCounter^®^ platform. 2 × 20 μm-thick whole tissue FFPE sections – including combined subfields and cortex/white matter - were used for RNA extraction. Bulk mRNA analysis was performed in the NanoString nSolver suite, with normalization of individual samples using six housekeeping genes included in the glial profiling panel. Benjamini-Yekutieli p-value adjustment for multiple comparisons was performed for differential expression. (**C**) Directed pathway scores in COVID hippocampus and MFG derived from the aforementioned nCounter^®^ glial profiling panel with positive, lighter bars indicating upregulation and negative, darker bars indicating downregulation. Pathways in both regions are compared against a baseline of Non-COVID and are arranged from most upregulated to most downregulated from top to bottom. Oligodendrocyte cell-type scores (specific to oligodendrocyte cells) (**D**) and markers (cell-type markers combined with some myelination-associated and functional genes) (**E**) derived from genes included in the glial profiling panel are significantly lower (Fig. 12F) in COVID patients as compared to Non-COVID patients. (**F**) Representative hippocampal IHC stains of Olig2 and PLP1 used for QuPath quantification in COVID (*N* = 11) and Non-COVID (*N* = 12) patients. IHC was performed in 5 μm-thick FFPE sections for both markers. Hippocampus Olig2 cells per mm^2^ (**G**) and PLP1 coverage (**H**) showed no differences at the whole region level but displayed some loss of Olig2 positivity in COVID patients at the subfield level (Fig. 12L). IHC comparisons were performed using an unpaired student’s t-test. Each group’s standard deviation is represented by error bars. “ns” = no significance, *p < 0.05, **p < 0.01, ***p < 0.001. Any comparisons lacking asterisks are non-significant. Scale bars = 100 μm

Seeking to evaluate potential alterations in the transcriptome of COVID patients, we performed bulk mRNA analysis using the Human Glial Profiling panel on the NanoString nCounter^®^ platform. Consecutive FFPE sections from Hippocampus/FG + ITG and MFG from the same patients in the COVID (*N* = 11, per region) and Non-COVID (*N* = 12, per region) groups were selected for analysis to examine regional transcriptomic differences at the extremes of the CP-proximity and AD axes. While disease-associated microglial and astrocytic pathways (“Stage 1 DAM”, “Stage 2 DAM”, “Microglia Neurodegenerative Phenotype (MGnD)”, “A1 Astrocyte”) (Fig. 10C) were only mildly upregulated in COVID (also see Table 4), we noticed a dysregulation in oligodendrocyte and myelogenesis systems (“Oligodendrocyte Markers”, “Oligodendrocyte DifferentiationMaturation”, and “Myelogenesis”) (Fig. 10C-E). Differently expressed genes (DEGs) within these pathways were downregulated in association with COVID-19 infection (Fig. 12C-E), suggestive of oligodendrocyte lineage dysfunction as a defining characteristic of the COVID tissue microenvironment. To evaluate these findings at the protein level, we performed IHC analysis of oligodendrocyte transcription factor 2 (Olig2), a protein marker for oligodendrocyte lineage cell types (Fig. 10F) [69–72]. Loss of Olig2-positive staining in COVID patients was observed within hippocampal subfields (Fig. 12L) but not at the whole-tissue level (Fig. 10G) and Olig2 positivity did not significantly correlate with plaque coverage (Supp. Figure 1S).

Previous literature has also identified impaired myelination as a potential upstream event and risk factor associated with AD pathogenesis [73]. Galactose-3-O-sulfotransferase 1 (GAL3ST1), also known as cerebroside sulfotransferase, is a significant enzyme involved in myelogenesis and is the final step in producing sulfatide, a glycosphingolipid representing around 30% of myelin lipids in combination with galactocerebroside [74, 75]. GAL3ST1 is the most significantly downregulated gene in COVID patients (Figs. 10A and 12G) and is of particular importance, with reduced CNS sulfatide levels potentially acting as a prodromal indicator of AD [75–77]. Seeking a general picture of myelin health in COVID patients, we performed IHC analysis of the percent area covered by proteolipid protein 1 (PLP1) (Fig. 10F) which, along with myelin basic protein, makes up 68% of myelin proteins [78]. No difference in PLP1 coverage was observed between groups in whole hippocampus (Fig. 10H) or subfields (Fig. 12M) and coverage did not correlate with plaque burden (Supp. Figure 1T), indicative of a more conserved myelin response to infection that was not primarily driven by Aβ pathology.

**Fig. 11:**
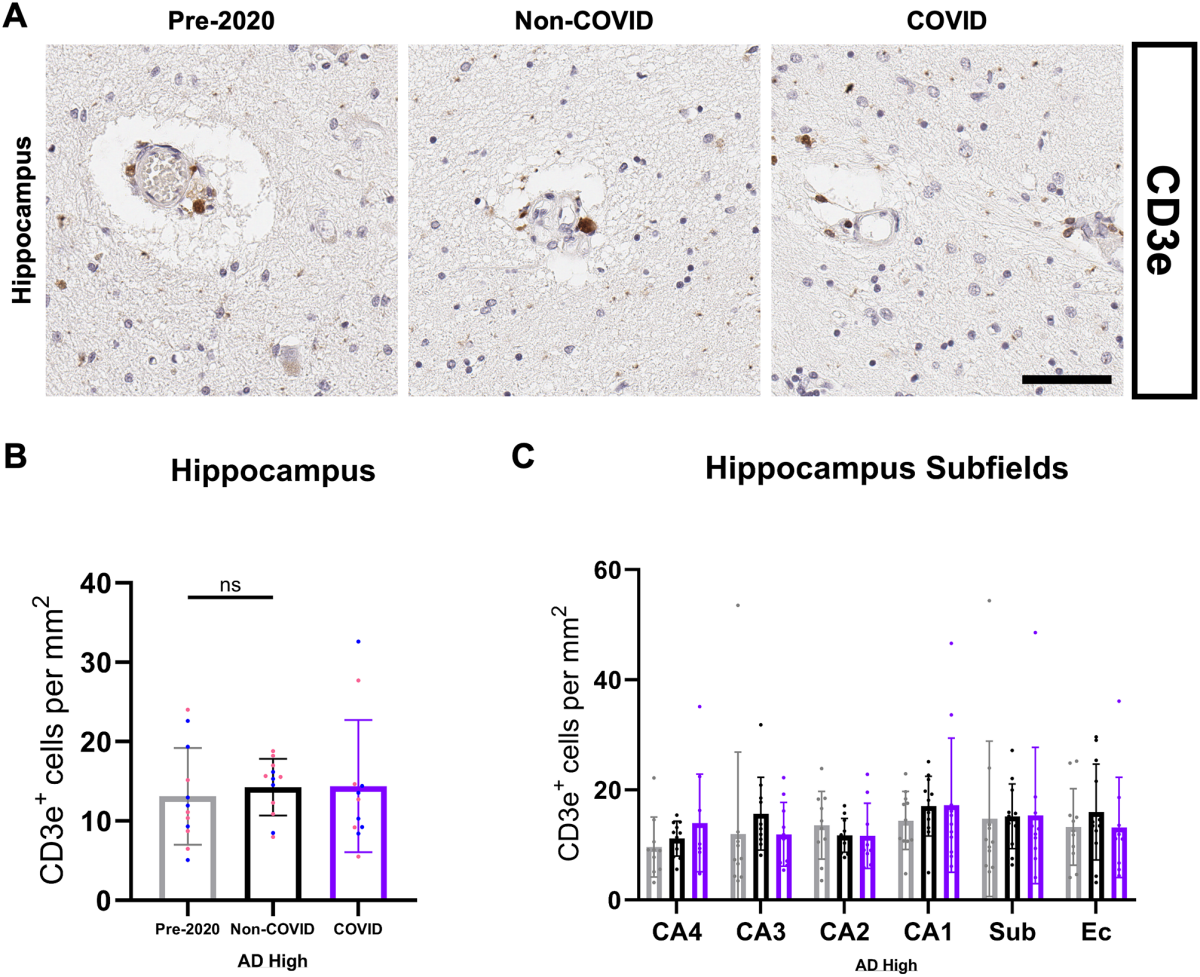
Quantification of CD3e-positive T-cells in the hippocampus CD3e-positive cells in the hippocampus were morphologically identical across groups, with a small, spherical soma (**A**). Most CD3e-positive cells were observed within the vasculature; however, some parenchymal infiltration could be observed, albeit in a limited manner. (**B**) Quantification of CD3e-positive T-cells in the hippocampus of COVID (*N* = 11), Non-COVID (*N* = 12), AND Pre-2020 (*N* = 12) patients. (**C**) Subfield quantification of CD3e-positive cell coverage in the hippocampus. Subfield comparisons were made using an ordinary two-way ANOVA with Tukey’s multiple comparisons test, with a single pooled variance. Each group’s standard deviation is represented by error bars. “ns” = no significance, *p < 0.05, **p < 0.01, ***p < 0.001. Any comparisons lacking asterisks are non-significant. Scale bar = 60 μm

**Fig. 12:**
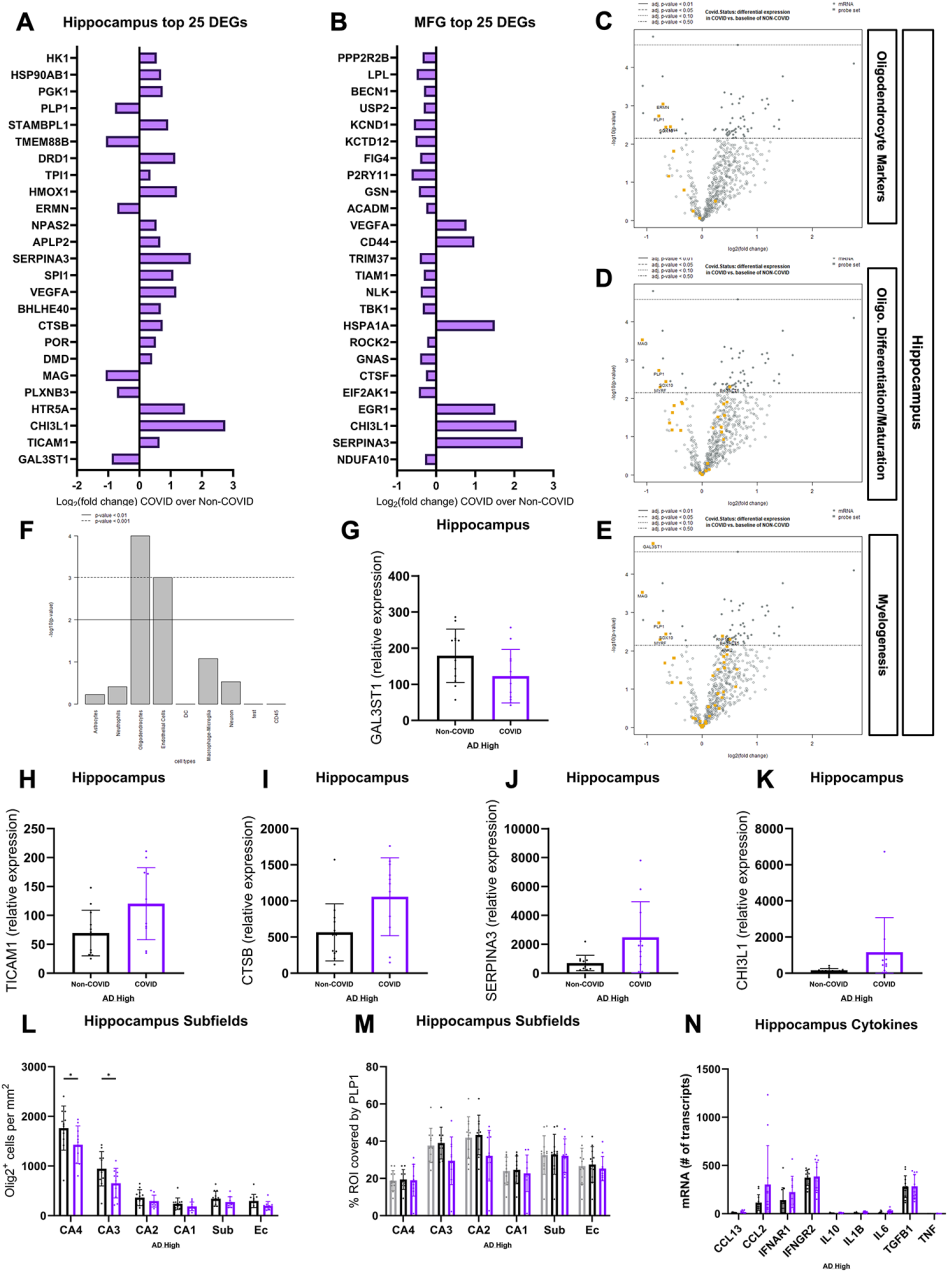
Bulk mRNA analysis of oligodendrocyte and myelin changes associated with COVID-AD. NanoString nCounter^®^ Bulk mRNA data analysis of whole FFPE tissue sections from the hippocampus and MFG of COVID (*N* = 11) vs. Non-COVID (*N* = 12) patients. The top 25 DEGs based on significance and their fold changes from hippocampus (**A**) and MFG (**B**) are listed from 1–25 with the most significant DEGs being located at the bottom of the graph. Volcano plots for the DEGs making up the Oligodendrocyte Markers (**C**), Oligodendrocyte Differentiation/Maturation (**D**), and Myelogenesis (**E**) pathways in the hippocampus with a baseline of Non-COVID. (**F**) p-values provided by nSolver for cell-type scoring validating oligodendrocyte scoring based on cell-type-specific DEG markers. (**G-K**) Univariate comparisons of selected DEGs. Adjusted p-values for individual genes can be found on Fig. 6 and a lack of stars does not indicate non-significance. Hippocampal subfield quantification of Olig2-positive cells per mm^2^ (**L**) and percent coverage of tissue by PLP1 (**M**). Comparisons performed using an ordinary two-way ANOVA with Tukey’s multiple comparisons test, with a single pooled variance and *p < 0.05, **p < 0.01, ***p < 0.001. Each group’s standard deviation is represented by error bars. **(N)** Univariate comparisons of nCounter transcript counts for selected cytokines. Generally, targets with visually lower counts such as CCL13, IL10, IL1B, 1L6, and TNF expressed too few transcripts for reliable comparisons to be made. Bulk mRNA analysis was performed in the NanoString nSolver suite, with normalization of individual samples using six housekeeping genes included in the glial profiling panel. Benjamini-Yekutieli p-value adjustment for multiple comparisons was performed for differential expression

TIR domain-containing adaptor molecule 1 (TICAM-1, also known as TRIF) is another DEG of interest, encoding an adaptor molecule to most toll-like receptors (TLR), some of which possess the capability to detect single-strand RNA viruses [79–81]. We demonstrate that COVID-19 patients possess subtly elevated TICAM-1 expression (Figs. 10A and 12H) and NF-kappaB signaling in the hippocampus (Fig. 10C), the latter of which is the direct effect of the TLR-TICAM-1 signaling cascade. Interested in determining whether the upregulation of these immune signaling pathways corresponded with increased T-cell surveillance and invasion of the hippocampal parenchyma in COVID patients, we performed IHC quantification of CD3e-positive cells to cover both CD8 + and CD4 + lineages (Fig. 11A). We did not observe a difference in the quantity of hippocampal T-cells across cohort groups (Fig. 11B-C), suggesting that the previously described neuroimmune changes are largely organized by innate, rather than adaptive, signaling cascades.

Compared to the hippocampus, the gene expression changes observed in the more CP-distant MFG were muted and less conserved between patients (Figs. 10A-B and 12A-B). Pathway differences in the MFG of COVID and Non-COVID patients were much less pronounced (Fig. 10C), and the oligodendrocyte lineage dysregulation observed in the hippocampus was absent (Fig. 10C). DEGs within the MFG were also more attenuated, with only a few astrocyte-associated genes (SERPINA3 and CHI3L1) overlapping with the profile observed in the hippocampus (Fig. 12B, J-K). Interestingly, astrocyte and pro/anti-inflammatory pathways (“A2 Astrocyte”, “Astrocyte Markers”, “Chemokines”, “Complement System”) were some of the upregulated systems amongst mostly neutral/downregulated pathways in the COVID MFG (Fig. 10C). Relative comparisons between cytokine transcripts included in the nCounter panel were analyzed, with trends towards increased CCL2 and IFNAR1 being observed in COVID patients (Fig. 12N).

## Conclusions

Sharing a strikingly similar at-risk population, COVID-19, AD, and their intersection represent a rapidly growing challenge for the world’s aging population. In this study, we conducted extensive immune and glial profiling of severe AD patients who survived systemic infection in the form of COVID-19 across multiple disease-relevant brain regions. While related, informative research has previously been performed on this topic [82], our data specifically focuses on the long-term effects of COVID-19 infection and recovery on neuroimmune states and potential progression of AD pathologies. In particular, we focused only on patients who did not die while experiencing a COVID-19 infection and who were COVID-negative at time of autopsy. Additionally, we were interested in the chronic neuroimmune alterations experienced by COVID patients and focussed on specific immunophenotypic shifts across cell types in the context of these two conditions.

Initial comparison of AD pathologies between Pre-2020, Non-COVID, and COVID patients revealed significant FG + ITG and EC elevation of Aβ pathological burden associated with COVID-19 at an average of 4.55 months post-infection. As one of the earliest brain structures affected by AD pathophysiology [83], EC parenchyma is already severely vulnerable to additional changes in the neural environment, potentially being ‘pushed over the edge’ into a more advanced disease state before other regions by elevated, periphery-originating inflammatory signals. Elevated burdens of AD pathological hallmarks were also localized within the grey matter layers of the hippocampus, a trend that is also reflected throughout our glial analyses. With a majority of AD pathological changes occurring within these layers, the suggestion that COVID-associated neurological insults are also localized here lends to the idea of it being involved in disease progression. Importantly, the regions experiencing the most significant increase in Aβ plaque coverage followed a pattern distinct from those of the glial population changes described below, suggesting that neuroimmune modulation could be both a primary effect of COVID-19 infection and upstream of any nascent accelerations of AD pathogenesis. Notably, milder trends towards increased Aβ burden were also observed in the more CP-proximal regions and hippocampal subfields of COVID patients in accordance with previous evidence that implicated the CP as a major conduit for the transmission of systemic inflammation to the CNS [14, 29]. The pathological profiles of the Pre-2020 and Non-COVID groups were similar and did not differ significantly from one another, addressing the possibility of asymptomatic and undiagnosed patients existing within the Non-COVID group by validating its representativeness against patients who could not have physically been infected by SARS-CoV-2.

As the quintessential immune cells of the brain, we first analyzed microglia using a variety of immunohistochemical markers to elucidate the precise neuroimmune profile of the COVID-AD brain. Systemic infection survival was significantly associated with chronically elevated numbers of hippocampal GM microglia, as evidenced by the quantification of Iba1-positive cells. As a pan-microglial marker [51], the increased amount of Iba1 positivity in COVID points to heightened microgliosis and immune activation as key characteristics of systemic-CNS immune communication, both of which have been previously demonstrated to occur in humans with AD and models of the disease [15, 84, 85]. Importantly, we show that the proliferation of microglia associated with COVID-19 infection is additive to the proliferation already present within AD-afflicted, Non-COVID patients. This refutes the possibility that because AD patients are already at an advanced state of neurodegeneration and inflammation, any accessory inflammatory insults would be essentially insignificant and negligible in the advancement of the disease. Complementary to this chronic elevation of proliferation, we observed a reduction in microglial homeostasis in COVID patients via quantification of P2RY12-positive cells. A marker for resting and ramified microglia [51], loss of P2RY12 positivity and morphological contraction of processes are well-documented indicators of neuroimmune activation, with reductions in this subpopulation in the parenchyma being some of the earliest microglial changes observed in AD [17]. Though also present in other brain regions, this shift from homeostatic to activated microglia was most acute in the CP-adjacent hippocampus and the nearby FG + ITG cortices, providing further evidence of significant CP involvement in the transmission of inflammatory signals. Taken in concert, inflammation from these individual diseases is cumulative, resulting in microglial populations that are less homoeostatic and more activated than those in severe AD alone. Additionally, the described changes in the neuroimmune axis occur despite a lack of SARS-Cov-2 positivity at time of autopsy and with an indeterminate cytokine profile, suggestive of an undetermined, intriguing mechanism by which this microglial profile is chronically altered in COVID patients.

Following the identification of increased microglial activation in COVID patients, we sought out a more nuanced image of the functional manifestations of this neuroimmune state. To do this, we evaluated various markers of microglial activity in our cohort groups, including CD68 and ferritin. No change in microglial phagocytosis and general reactivity as assessed by CD68 immunopositivity [51, 86] was observed in the COVID group, however, a trend towards elevated ferritin-positivity was present in the GM of all assessed brain regions. Though subtle, this trend suggests that COVID patients tend to have more dystrophic, iron-accumulating microglia [57, 59], possibly as a result of chronic metabolic stress and ‘burnout’ [31, 87] or, more intriguingly, in response to increased levels of periphery-originating free iron in the brain associated with COVID-19 infection and resultant sequelae [88]. The activation state of CNS microglia in COVID patients was largely indeterminate, and further investigation is required concerning the functional characteristics of this population.

AD patients with COVID-19 also presented with extensive hippocampal astrocyte dysregulation as compared to their Non-COVID counterparts exhibited by our quantifications of GFAP and ALDH1L1. While literature concerning the presence and effect of GFAP-positive reactive astrogliosis in humans with AD remains largely incohesive, studies performed in disease models have shown that experimentally attenuating reactive astrocyte proliferation results in the advancement of Aβ pathology [89], highlighting our observation of chronically reduced GFAP-positive astrocytes in COVID patients as a potential avenue for AD advancement. We also observed an attenuated but similar reduction in ALDH1L1-positive astrocytes, suggestive of a global effect of systemic infection on the astrocyte lineage rather than being limited to a reactive subtype.

The indiscriminate nature of COVID astrocyte dysfunction and the cell type’s crucial role in the neurovascular unit [90] also imply some level of BBB dysfunction in these patients, potentially amplifying communication of systemic inflammation to the CNS. This is further supported by previous reports of BBB changes existing in COVID-19 and AD separately [91, 92]. This hypothesis is also in line with our ferritin-positive microglia data, as destabilization in CNS iron homeostasis follows BBB breakdown in multiple forms of disease [93]. Despite this information, we did not observe focal astrocytic loss in proximity to blood vessels in COVID patients, which could be indicative of other forms of BBB insult, such as the dysregulation of gap junctions and increased permeability between CNS and periphery. Further investigation is necessary to better characterize if and to what extent the BBB plays a role in the changes observed in AD patients suffering from systemic infection.

Interestingly, acute phenotypic shifts in astrocytes were only observed in CP-adjacent regions such as the hippocampus, which suggests that astrocyte dysfunction and/or loss in COVID patients is more associated with changes at the blood-cerebrospinal fluid barrier (BCSFB) rather than at the BBB and general vasculature throughout the brain parenchyma. As we have observed astrocytic loss to not be significantly tied to vessel and BBB proximity, it is possible that the BCSFB is the major effector in inflammatory signaling and dysregulation as the other major CNS interface with the periphery. Further research is required to better distinguish hippocampal and CP-proximal astrocytes from their counterparts in the MFG and other regions in COVID patients, and to understand the implications of these shifts on the neuroimmune response to COVID and AD. Aside from vasculature support, CNS astrocytes have robust roles in synaptic maintenance and the perpetuation of the neuroimmune response [94], and more specific functional characterization of COVID astrocytes should be performed in the future to understand their potential impact on the progression of AD pathology.

Whole-tissue transcriptomic analysis of the same patients generally confirmed the observations made in our immunohistochemical studies but also revealed the presence of extensive oligodendrocyte lineage (OL) dysregulation in COVID patients. WM and OL pathway downregulation have been extensively documented in COVID-19 patients and experimental models [95–97], and the detection of these changes in AD patients highlights another infection-associated risk factor for disease progression considering existing literature placing myelin dysfunction as an early change in AD patients [73, 76, 77, 98]. The lack of agreement in these changes at the protein level as assessed by the more broadly labeling markers OLIG2 and PLP1 could reflect a more particularized alteration in these pathways, such as dysregulation in a subset of OL cells or in the lipid composition of the myelin sheath instead of the lipid-protein profile. Since we performed whole tissue RNA analysis, we did not have the spatial resolution for these changes in expression to determine if they were preferentially localized to the cortex or WM. Furthermore, many of the changes observed in glia-related pathways in the hippocampal subfields were localized to the directly periventricular CA4-CA2 regions, implying that these regions receive the highest concentration of inflammatory signals from the CP before diffusing to the rest of the hippocampus. This was also supported by the degree of expression changes between the MFG and the hippocampus, with the latter experiencing more acute modulations of DEGs, pathways, and overall tissue function.

Bulk RNA-seq also further validated patients in the COVID group as being previously infected, with individual genes upregulated in the group such as TICAM1 and CTSB (Supp. Fig. 4I) having previously been demonstrated to be involved in COVID-19 pathogenesis [99–101].

Interestingly, the most conserved changes in the top 25 DEGs between regions were those associated with astrocytes such as ‘CHI3L1’ and ‘SERPINA3’, again suggesting the presence of CNS-wide BBB dysregulation which is additive to blood-CSF barrier dysfunction in the hippocampus resulting in differing degrees of neuroimmune modulation. CHI3L1 (also known as YKL40) is of particular interest due to emerging evidence highlighting its role in neuroinflammatory astrocytic mechanisms, including the impairment of neurogenesis and the development of cognitive impairment [102, 103]. With upregulation of the gene being observed both near and far from the CP (albeit, at different intensities), it’s plausible that the astrocytic phenotype is associated with BBB dysregulation and, with recent studies in animal models tying its activity to Aβ burden [104], it could be an avenue for the advancement in Aβ pathology observed in COVID-AD patients.

Here we demonstrate the ability for COVID-associated systemic inflammatory insults to be communicated to the CNS across multiple interfaces, inducing a pro-inflammatory neuroimmune environment. In patients already suffering from AD, this inflammation is cumulative, adding to pre-existing disruptions of microglia and brain homeostasis associated with the disease, potentially increasing the risk for disease advancement. Novel alterations in astrocyte populations and oligodendrocyte function were also observed, conveying the possible extent to which peripherally located stimuli can influence the cellular composition of the CNS in AD and other neurodegenerative diseases.

While the self-report of COVID-19 infection history by patients is a limitation of the study, we attempted to address this by adding comparisons with non-infected pre-2020 patients, which validated our non-COVID control group and allows for important insights into the interaction between systemic infection and AD pathology to be taken from this study. Previous studies performed by our group [20, 31] have investigated the microglial response to Alzheimer’s disease pathology in comparison to non-demented control patients. This study focused on the effect of a COVID-19 infection on the neuroimmune environment with concomitant Alzheimer’s disease pathology. While this study would have benefitted from including both COVID-negative and more importantly COVID-positive non-demented control patient cohorts, brains from donors with the latter condition were not available in our brain bank at the time the study was conducted. It will be very important to continue to study the effects of severe systemic infections, including COVID-19 and more general sepsis on the neuroimmune environment in brains from neurologically normal donors and donors suffering from neurodegenerative conditions. Despite limitations in the power of our study, significant changes in the CNS environment could still be detected, and we encourage other centers to perform similar inquiries into COVID patients at their banks as further validation of these findings will only help to accelerate our understanding of the interaction between these two diseases and could potentially inform future therapeutic strategies.

## Data Availability

The datasets used and/or analyzed during the current study are available from the corresponding author on reasonable request.

## Abbreviations

AD: Alzheimer’s disease
Aβ: Amyloid beta
NFT: Neurofibrillary tangles
CNS: Central nervous system
IHC: Immunohistochemistry
BBB: Blood-brain barrier
CP: Choroid plexus
ADNC: Alzheimer’s disease neuropathological changes
PMI: Post-mortem interval
FFPE: Formalin-fixed paraffin-embedded
CA: Cornus ammonis
MFG: Medial frontal gyrus
STG: Superior temporal gyrus
FG: Fusiform gyrus
ITG: Inferior temporal gyrus
WM: White matter
SUB: Subiculum
EC: Entorhinal cortex
Iba1: Ionized calcium-binding adaptor molecule 1
P2RY12: Purinergic receptor P2Y12
CD68: Cluster of differentiation 68
GFAP: Glial fibrillary acidic protein
ALDH1L1: Aldehyde dehydrogenase 1 family member L1
DEG: Differently expressed genes
Olig2: Oligodendrocyte transcription factor 2
GAL3ST1: Galactose-3-O-sulfotransferase 1
PLP1: Proteolipid protein 1
TLR: Toll-like receptors
TICAM-1: TIR domain containing adaptor molecule 1
OL: Oligodendrocyte lineage
BCSFB: Blood-cerebrospinal fluid barrier

## References

[1] Walker KA, Ficek BN, Westbrook R. Understanding the role of systemic inflammation in Alzheimer’s Disease. ACS Chem Neurosci. 2019;10(8):3340–2. 31241312 10.1021/acschemneuro.9b00333

[2] Holmes C, El-Okl M, Williams AL, Cunningham C, Wilcockson D, Perry VH. Systemic infection, interleukin 1β, and cognitive decline in Alzheimer’s disease. J Neurol Neurosurg Psychiatry. 2003;74(6):788–9. 12754353 10.1136/jnnp.74.6.788 PMC1738504

[3] Asby D, Boche D, Allan S, Love S, Miners JS. Systemic infection exacerbates cerebrovascular dysfunction in Alzheimer’s disease. Brain. 2021;144(6):1869–83. 33723589 10.1093/brain/awab094 PMC8320299

[4] Teeling JL, Perry VH. Systemic infection and inflammation in acute CNS injury and chronic neurodegeneration: underlying mechanisms. Neuroscience. 2009;158(3):1062–73. 18706982 10.1016/j.neuroscience.2008.07.031

[5] Rakic S, Hung YMA, Smith M, So D, Tayler HM, Varney W, et al. Systemic infection modifies the neuroinflammatory response in late stage Alzheimer’s disease. Acta Neuropathol Commun. 2018;6(1):88. 30193587 10.1186/s40478-018-0592-3 PMC6127939

[6] Chou CH, Lee JT, Lin CC, Sung YF, Lin CC, Muo CH, et al. Septicemia is associated with increased risk for dementia: a population-based longitudinal study. Oncotarget. 2017;8(48):84300–8. 29137424 10.18632/oncotarget.20899 PMC5663596

[7] Sartori AC, Vance DE, Slater LZ, Crowe M. The impact of inflammation on cognitive function in older adults: implications for Health Care Practice and Research. J Neurosci Nurs. 2012;44(4):206–17. 22743812 10.1097/JNN.0b013e3182527690 PMC3390758

[8] Hotchkiss RS, Moldawer LL, Opal SM, Reinhart K, Turnbull IR, Vincent JL. Sepsis and septic shock. Nat Rev Dis Primers. 2016;2:16045. 28117397 10.1038/nrdp.2016.45 PMC5538252

[9] Ramos-Casals M, Brito-Zerón P, Mariette X. Systemic and organ-specific immune-related manifestations of COVID-19. Nat Rev Rheumatol. 2021;17(6):315–32. 33903743 10.1038/s41584-021-00608-z PMC8072739

[10] Kalil AC, Thomas PG. Influenza virus-related critical illness: pathophysiology and epidemiology. Crit Care. 2019;23:258. 31324202 10.1186/s13054-019-2539-x PMC6642581

[11] Xu E, Xie Y, Al-Aly Z. Long-term neurologic outcomes of COVID-19. Nat Med. 2022;28(11):2406–15. 36138154 10.1038/s41591-022-02001-z PMC9671811

[12] COVID-19 epidemiological update. – 15 March 2024 [Internet]. [cited 2024 Apr 6]. https://www.who.int/publications/m/item/covid-19-epidemiological-update-15-march-2024

[13] Nouraeinejad A. Brain fog as a long-term Sequela of COVID-19. SN Compr Clin Med. 2023;5(1):9. 36466122 10.1007/s42399-022-01352-5 PMC9685075

[14] Yang AC, Kern F, Losada PM, Agam MR, Maat CA, Schmartz GP, et al. Dysregulation of brain and choroid plexus cell types in severe COVID-19. Nature. 2021;595(7868):565–71. 34153974 10.1038/s41586-021-03710-0 PMC8400927

[15] Colonna M, Butovsky O. Microglia function in the Central Nervous System during Health and Neurodegeneration. Annu Rev Immunol. 2017;35:441–68. 28226226 10.1146/annurev-immunol-051116-052358 PMC8167938

[16] Poloni TE, Medici V, Moretti M, Visonà SD, Cirrincione A, Carlos AF, et al. COVID-19‐related neuropathology and microglial activation in elderly with and without dementia. Brain Pathol. 2021;31(5):e12997. 34145669 10.1111/bpa.12997 PMC8412067

[17] Walker DG, Tang TM, Mendsaikhan A, Tooyama I, Serrano GE, Sue LI, et al. Patterns of expression of purinergic receptor P2RY12, a putative marker for non-activated microglia, in aged and Alzheimer’s Disease brains. Int J Mol Sci. 2020;21(2):678. 31968618 10.3390/ijms21020678 PMC7014248

[18] Keren-Shaul H, Spinrad A, Weiner A, Matcovitch-Natan O, Dvir-Szternfeld R, Ulland TK, et al. A Unique Microglia Type Associated with Restricting Development of Alzheimer’s Disease. Cell. 2017;169(7):1276–e129017. 28602351 10.1016/j.cell.2017.05.018

[19] Maeda J, Minamihisamatsu T, Shimojo M, Zhou X, Ono M, Matsuba Y, et al. Distinct microglial response against Alzheimer’s amyloid and tau pathologies characterized by P2Y12 receptor. Brain Commun. 2021;3(1):fcab011. 33644757 10.1093/braincomms/fcab011 PMC7901060

[20] Prokop S, Miller KR, Labra SR, Pitkin RM, Hoxha K, Narasimhan S, et al. Impact of TREM2 risk variants on brain region-specific immune activation and plaque microenvironment in Alzheimer’s disease patient brain samples. Acta Neuropathol. 2019;138(4):613–30. 31350575 10.1007/s00401-019-02048-2 PMC6939638

[21] Chen Y, Klein SL, Garibaldi BT, Li H, Wu C, Osevala NM, et al. Aging in COVID-19: vulnerability, immunity and intervention. Ageing Res Rev. 2021;65:101205. 33137510 10.1016/j.arr.2020.101205 PMC7604159

[22] Rabinovici GD. Late-onset Alzheimer Disease. Continuum (Minneap Minn). 2019;25(1):14–33. 30707185 10.1212/CON.0000000000000700 PMC6548536

[23] Liddelow SA, Barres BA. Reactive astrocytes: production, function, and therapeutic potential. Immunity. 2017;46(6):957–67. 28636962 10.1016/j.immuni.2017.06.006

[24] Liddelow SA, Guttenplan KA, Clarke LE, Bennett FC, Bohlen CJ, Schirmer L, et al. Neurotoxic reactive astrocytes are induced by activated microglia. Nature. 2017;541(7638):481–7. 28099414 10.1038/nature21029 PMC5404890

[25] Liu C, Zhao XM, Wang Q, Du TT, Zhang MX, Wang HZ, et al. Astrocyte-derived SerpinA3N promotes neuroinflammation and epileptic seizures by activating the NF-κB signaling pathway in mice with temporal lobe epilepsy. J Neuroinflamm. 2023;20(1):161. 10.1186/s12974-023-02840-8 PMC10329806 37422673

[26] Kraft AW, Hu X, Yoon H, Yan P, Xiao Q, Wang Y, et al. Attenuating astrocyte activation accelerates plaque pathogenesis in APP/PS1 mice. FASEB J. 2013;27(1):187–98. 23038755 10.1096/fj.12-208660 PMC3528309

[27] Abbott NJ, Rönnbäck L, Hansson E. Astrocyte–endothelial interactions at the blood–brain barrier. Nat Rev Neurosci. 2006;7(1):41–53. 16371949 10.1038/nrn1824

[28] Daneman R, Prat A. The blood–brain barrier. Cold Spring Harb Perspect Biol. 2015;7(1):a020412. 25561720 10.1101/cshperspect.a020412 PMC4292164

[29] Pellegrini L, Albecka A, Mallery DL, Kellner MJ, Paul D, Carter AP, et al. SARS-CoV-2 infects the Brain Choroid Plexus and disrupts the Blood-CSF barrier in human brain organoids. Cell Stem Cell. 2020;27(6):951–e9615. 33113348 10.1016/j.stem.2020.10.001 PMC7553118

[30] Suzzi S, Tsitsou-Kampeli A, Schwartz M. The type I interferon antiviral response in the choroid plexus and the cognitive risk in COVID-19. Nat Immunol. 2023;24(2):220–4. 36717725 10.1038/s41590-022-01410-z

[31] Bathe T, Hery GP, Villareal JAB, Phillips JL, Cohen EM, Sharma RV, et al. Disease and brain region specific immune response profiles in neurodegenerative diseases with pure and mixed protein pathologies. Acta Neuropathol Commun. 2024;12(1):54. 38581050 10.1186/s40478-024-01770-7 PMC10996248

[32] Montine TJ, Phelps CH, Beach TG, Bigio EH, Cairns NJ, Dickson DW, et al. National Institute on Aging–Alzheimer’s Association guidelines for the neuropathologic assessment of Alzheimer’s disease: a practical approach. Acta Neuropathol. 2012;123(1):1–11. 22101365 10.1007/s00401-011-0910-3 PMC3268003

[33] Hyman BT, Phelps CH, Beach TG, Bigio EH, Cairns NJ, Carrillo MC, et al. National Institute on Aging–Alzheimer’s Association guidelines for the neuropathologic assessment of Alzheimer’s disease. Alzheimer’s Dement. 2012;8(1):1–13. 22265587 10.1016/j.jalz.2011.10.007 PMC3266529

[34] McKeith IG, Boeve BF, Dickson DW, Halliday G, Taylor JP, Weintraub D, et al. Diagnosis and management of dementia with Lewy bodies. Neurology. 2017;89(1):88–100. 28592453 10.1212/WNL.0000000000004058 PMC5496518

[35] Nelson PT, Dickson DW, Trojanowski JQ, Jack CR, Boyle PA, Arfanakis K, et al. Limbic-predominant age-related TDP-43 encephalopathy (LATE): consensus working group report. Brain. 2019;142(6):1503–27. 31039256 10.1093/brain/awz099 PMC6536849

[36] Cykowski MD, Arumanayagam AS, Powell SZ, Rivera AL, Abner EL, Roman GC, et al. Patterns of amygdala region pathology in LATE-NC: subtypes that differ with regard to TDP-43 histopathology, genetic risk factors, and comorbid pathologies. Acta Neuropathol. 2022;143(5):531–45. 35366087 10.1007/s00401-022-02416-5 PMC9038848

[37] Vonsattel JPG, Myers RH, Tessa Hedley-Whyte E, Ropper AH, Bird ED, Richardson EP Jr. Cerebral amyloid angiopathy without and with cerebral hemorrhages: a comparative histological study. Ann Neurol. 1991;30(5):637–49. 1763890 10.1002/ana.410300503

[38] Kovacs GG, Ferrer I, Grinberg LT, Alafuzoff I, Attems J, Budka H, et al. Aging-related tau astrogliopathy (ARTAG): harmonized evaluation strategy. Acta Neuropathol. 2016;131(1):87–102. 26659578 10.1007/s00401-015-1509-x PMC4879001

[39] Tsering W, Hery GP, Phillips JL, Lolo K, Bathe T, Villareal JA, et al. Transformation of non-neuritic into neuritic plaques during AD progression drives cortical spread of tau pathology via regenerative failure. Acta Neuropathol Commun. 2023;11(1):190. 38037144 10.1186/s40478-023-01688-6 PMC10691154

[40] Bankhead P, Loughrey MB, Fernández JA, Dombrowski Y, McArt DG, Dunne PD, et al. QuPath: open source software for digital pathology image analysis. Sci Rep. 2017;7(1):16878. 29203879 10.1038/s41598-017-17204-5 PMC5715110

[41] Zindy E. add_rings.groovy concentric circles script [Internet]. DIAPath, Centre for Microscopy and Molecular Imaging, Université Libre de Bruxelles (ULB), Gosselies, Belgium; [cited 2024 Jul 6]. https://github.com/diapath/qupath_scripts

[42] Schmidt U, Weigert M, Broaddus C, Myers G. Cell detection with Star-Convex polygons. In: Frangi AF, Schnabel JA, Davatzikos C, Alberola-López C, Fichtinger G, editors. Medical Image Computing and Computer assisted intervention – MICCAI 2018. Cham: Springer International Publishing; 2018. pp. 265–73.

[43] ROCHE - eLabDoc [Internet]. [cited 2024 May 13]. https://elabdoc-prod.roche.com/eLD/web/pi/en/products/3.6.8.50.1.1

[44] NanoString University [Internet]. [cited 2024 May 17]. Preparing RNA for nCounter Assays (FFPE or Fresh Frozen Samples). https://university.nanostring.com/preparing-rna-for-ncounter-assays-ffpe-or-fresh-frozen-samples

[45] NanoString. University [Internet]. [cited 2024 May 13]. Quick Guide: nCounter MAX/FLEX. https://university.nanostring.com/ncounter-maxflex-system-quick-start-guide

[46] Scelsi CL, Rahim TA, Morris JA, Kramer GJ, Gilbert BC, Forseen SE. The Lateral Ventricles: A Detailed Review of Anatomy, Development, and Anatomic Variations. American Journal of Neuroradiology [Internet]. 2020 Feb 20 [cited 2024 May 16]; https://www.ajnr.org/content/early/2020/02/20/ajnr.A6456 10.3174/ajnr.A6456 PMC7144651 32079598

[47] Ferrarini L, Palm WM, Olofsen H, van Buchem MA, Reiber JHC, Admiraal-Behloul F. Shape differences of the brain ventricles in Alzheimer’s disease. NeuroImage. 2006;32(3):1060–9. 16839779 10.1016/j.neuroimage.2006.05.048

[48] Duffner F, Schiffbauer H, Glemser D, Skalej M, Freudenstein D. Anatomy of the cerebral ventricular system for endoscopic neurosurgery: a magnetic resonance study. Acta Neurochir. 2003;145(5):359–68. 12820042 10.1007/s00701-003-0021-6

[49] Levites Y, Das P, Price RW, Rochette MJ, Kostura LA, McGowan EM, et al. Anti-Aβ_42_– and anti-Aβ_40_–specific mAbs attenuate amyloid deposition in an Alzheimer disease mouse model. J Clin Invest. 2006;116(1):193–201. 16341263 10.1172/JCI25410 PMC1307561

[50] Xia Y, Prokop S, Gorion KMM, Kim JD, Sorrentino ZA, Bell BM, et al. Tau Ser208 phosphorylation promotes aggregation and reveals neuropathologic diversity in Alzheimer’s disease and other tauopathies. Acta Neuropathol Commun. 2020;8(1):88. 32571418 10.1186/s40478-020-00967-w PMC7310041

[51] Hopperton KE, Mohammad D, Trépanier MO, Giuliano V, Bazinet RP. Markers of microglia in post-mortem brain samples from patients with Alzheimer’s disease: a systematic review. Mol Psychiatry. 2018;23(2):177–98. 29230021 10.1038/mp.2017.246 PMC5794890

[52] Illes P, Rubini P, Ulrich H, Zhao Y, Tang Y. Regulation of Microglial functions by Purinergic mechanisms in the healthy and diseased CNS. Cells. 2020;9(5):1108. 32365642 10.3390/cells9051108 PMC7290360

[53] Butovsky O, Jedrychowski MP, Moore CS, Cialic R, Lanser AJ, Gabriely G, et al. Identification of a unique TGF-β dependent molecular and functional signature in Microglia. Nat Neurosci. 2014;17(1):131–43. 24316888 10.1038/nn.3599 PMC4066672

[54] Chistiakov DA, Killingsworth MC, Myasoedova VA, Orekhov AN, Bobryshev YV. CD68/macrosialin: not just a histochemical marker. Lab Invest. 2017;97(1):4–13. 27869795 10.1038/labinvest.2016.116

[55] Holness CL, Simmons DL. Molecular cloning of CD68, a human macrophage marker related to lysosomal glycoproteins. Blood. 1993;81(6):1607–13. 7680921 10.1182/blood.V81.6.1607.1607

[56] McCarthy RC, Sosa JC, Gardeck AM, Baez AS, Lee CH, Wessling-Resnick M. Inflammation-induced iron transport and metabolism by brain microglia. J Biol Chem. 2018;293(20):7853–63. 29610275 10.1074/jbc.RA118.001949 PMC5961037

[57] Kenkhuis B, Somarakis A, de Haan L, Dzyubachyk O, IJsselsteijn ME, de Miranda NFCC, et al. Iron loading is a prominent feature of activated microglia in Alzheimer’s disease patients. Acta Neuropathol Commun. 2021;9(1):27. 33597025 10.1186/s40478-021-01126-5 PMC7887813

[58] Levi S, Ripamonti M, Moro AS, Cozzi A. Iron imbalance in neurodegeneration. Mol Psychiatry. 2024;1–14. 10.1038/s41380-023-02399-z PMC11176077 38212377

[59] Lopes KO, Sparks DL, Streit WJ. Microglial dystrophy in the aged and Alzheimer’s disease brain is associated with ferritin immunoreactivity. Glia. 2008;56(10):1048–60. 18442088 10.1002/glia.20678

[60] Simpson JE, Ince PG, Lace G, Forster G, Shaw PJ, Matthews F, et al. Astrocyte phenotype in relation to Alzheimer-type pathology in the ageing brain. Neurobiol Aging. 2010;31(4):578–90. 18586353 10.1016/j.neurobiolaging.2008.05.015

[61] Zhang Z, Ma Z, Zou W, Guo H, Liu M, Ma Y, et al. The appropriate marker for astrocytes: comparing the distribution and expression of three astrocytic markers in different mouse cerebral regions. Biomed Res Int. 2019;2019:9605265. 31341912 10.1155/2019/9605265 PMC6613026

[62] Kim KY, Shin KY, Chang KA. GFAP as a potential biomarker for Alzheimer’s Disease: a systematic review and Meta-analysis. Cells. 2023;12(9):1309. 37174709 10.3390/cells12091309 PMC10177296

[63] Barres BA. The mystery and magic of Glia: a perspective on their roles in Health and Disease. Neuron. 2008;60(3):430–40. 18995817 10.1016/j.neuron.2008.10.013

[64] Forrest SL, Kim JH, Crockford DR, Huynh K, Cheong R, Knott S, et al. Distribution patterns of astrocyte populations in the human cortex. Neurochem Res. 2023;48(4):1222–32. 35930103 10.1007/s11064-022-03700-2 PMC10030423

[65] Cahoy JD, Emery B, Kaushal A, Foo LC, Zamanian JL, Christopherson KS, et al. A transcriptome database for astrocytes, neurons, and oligodendrocytes: a New Resource for understanding Brain development and function. J Neurosci. 2008;28(1):264–78. 18171944 10.1523/JNEUROSCI.4178-07.2008 PMC6671143

[66] Perez-Nievas BG, Serrano-Pozo A. Deciphering the astrocyte reaction in Alzheimer’s Disease. Front Aging Neurosci. 2018;10:114. 29922147 10.3389/fnagi.2018.00114 PMC5996928

[67] Sekar S, McDonald J, Cuyugan L, Aldrich J, Kurdoglu A, Adkins J, et al. Alzheimer’s disease is associated with altered expression of genes involved in immune response and mitochondrial processes in astrocytes. Neurobiol Aging. 2015;36(2):583–91. 25448601 10.1016/j.neurobiolaging.2014.09.027 PMC4315763

[68] Lertkiatmongkol P, Liao D, Mei H, Hu Y, Newman PJ. Endothelial functions of platelet/endothelial cell adhesion molecule-1 (CD31). Curr Opin Hematol. 2016;23(3):253. 27055047 10.1097/MOH.0000000000000239 PMC4986701

[69] Zhou Q, Wang S, Anderson DJ. Identification of a Novel Family of Oligodendrocyte Lineage-Specific Basic Helix–Loop–Helix transcription factors. Neuron. 2000;25(2):331–43. 10719889 10.1016/S0896-6273(00)80898-3

[70] Ligon KL, Fancy SPJ, Franklin RJM, Rowitch DH. Olig gene function in CNS development and disease. Glia. 2006;54(1):1–10. 16652341 10.1002/glia.20273

[71] Zhou Q, Anderson DJ. The bHLH transcription factors OLIG2 and OLIG1 couple neuronal and glial subtype specification. Cell. 2002;109(1):61–73. 11955447 10.1016/S0092-8674(02)00677-3

[72] Rowitch DH, Lu QR, Kessaris N, Richardson WD. An ‘oligarchy’ rules neural development. Trends Neurosci. 2002;25(8):417–22. 12127759 10.1016/S0166-2236(02)02201-4

[73] Depp C, Sun T, Sasmita AO, Spieth L, Berghoff SA, Nazarenko T, et al. Myelin dysfunction drives amyloid-β deposition in models of Alzheimer’s disease. Nature. 2023;618(7964):349–57. 37258678 10.1038/s41586-023-06120-6 PMC10247380

[74] Poitelon Y, Kopec AM, Belin S. Myelin Fat facts: an overview of lipids and fatty acid metabolism. Cells. 2020;9(4):812. 32230947 10.3390/cells9040812 PMC7226731

[75] Jeon SB, Yoon HJ, Park SH, Kim IH, Park EJ, Sulfatide. A major lipid component of myelin sheath, activates inflammatory responses as an endogenous stimulator in Brain-Resident Immune cells. J Immunol. 2008;181(11):8077–87. 10.4049/jimmunol.181.11.8077 19018000

[76] Han X, Holtzman M, McKeel D, Kelley DW, Morris J. Substantial sulfatide deficiency and ceramide elevation in very early Alzheimer’s disease: potential role in disease pathogenesis. J Neurochem. 2002;82(4):809–18. 12358786 10.1046/j.1471-4159.2002.00997.x

[77] Qiu S, Palavicini JP, Wang J, Gonzalez NS, He S, Dustin E, et al. Adult-onset CNS myelin sulfatide deficiency is sufficient to cause Alzheimer’s disease-like neuroinflammation and cognitive impairment. Mol Neurodegeneration. 2021;16(1):64. 10.1186/s13024-021-00488-7 PMC8442347 34526055

[78] Ruskamo S, Raasakka A, Pedersen JS, Martel A, Škubník K, Darwish T, et al. Human myelin proteolipid protein structure and lipid bilayer stacking. Cell Mol Life Sci. 2022;79(8):419. 35829923 10.1007/s00018-022-04428-6 PMC9279222

[79] Khanmohammadi S, Rezaei N. Role of toll-like receptors in the pathogenesis of COVID‐19. J Med Virol. 2021;93(5):2735–9. 33506952 10.1002/jmv.26826 PMC8014260

[80] Oshiumi H, Matsumoto M, Funami K, Akazawa T, Seya T. TICAM-1, an adaptor molecule that participates in toll-like receptor 3–mediated interferon-β induction. Nat Immunol. 2003;4(2):161–7. 12539043 10.1038/ni886

[81] Walker DG, Tang TM, Lue LF. Increased expression of toll-like receptor 3, an Anti-viral Signaling Molecule, and related genes in Alzheimer’s Disease brains. Exp Neurol. 2018;309:91–106. 30076830 10.1016/j.expneurol.2018.07.016 PMC6151184

[82] Granholm ACE, Englund E, Gilmore A, Head E, Yong WH, Perez SE, et al. Neuropathological findings in Down syndrome, Alzheimer’s disease and control patients with and without SARS-COV-2: preliminary findings. Acta Neuropathol. 2024;147(1):92. 38801558 10.1007/s00401-024-02743-9 PMC11130011

[83] Igarashi KM. Entorhinal cortex dysfunction in Alzheimer’s disease. Trends Neurosci. 2023;46(2):124–36. 36513524 10.1016/j.tins.2022.11.006 PMC9877178

[84] Butovsky O, Weiner HL. Microglial signatures and their role in health and disease. Nat Rev Neurosci. 2018;19(10):622–35. 30206328 10.1038/s41583-018-0057-5 PMC7255106

[85] Hansen DV, Hanson JE, Sheng M. Microglia in Alzheimer’s disease. J Cell Biol. 2018;217(2):459–72. 29196460 10.1083/jcb.201709069 PMC5800817

[86] Jurga AM, Paleczna M, Kuter KZ. Overview of General and discriminating markers of Differential Microglia phenotypes. Front Cell Neurosci. 2020;14:198. 32848611 10.3389/fncel.2020.00198 PMC7424058

[87] Kenkhuis B, van Eekeren M, Parfitt DA, Ariyurek Y, Banerjee P, Priller J, et al. Iron accumulation induces oxidative stress, while depressing inflammatory polarization in human iPSC-derived microglia. Stem Cell Rep. 2022;17(6):1351–65. 10.1016/j.stemcr.2022.04.006 PMC9213827 35523178

[88] Duca L, Ottolenghi S, Coppola S, Rinaldo R, Dei Cas M, Rubino FM, et al. Differential Redox State and Iron Regulation in Chronic Obstructive Pulmonary Disease, Acute Respiratory Distress Syndrome and Coronavirus Disease 2019. Antioxid (Basel). 2021;10(9):1460. 10.3390/antiox10091460 PMC8470076 34573092

[89] Pekny M, Pekna M. Astrocyte reactivity and reactive astrogliosis: costs and benefits. Physiol Rev. 2014;94(4):1077–98. 25287860 10.1152/physrev.00041.2013

[90] Schaeffer S, Iadecola C. Revisiting the neurovascular unit. Nat Neurosci. 2021;24(9):1198–209. 34354283 10.1038/s41593-021-00904-7 PMC9462551

[91] Greene C, Connolly R, Brennan D, Laffan A, O’Keeffe E, Zaporojan L et al. Blood–brain barrier disruption and sustained systemic inflammation in individuals with long COVID-associated cognitive impairment. Nat Neurosci. 2024;1–12. 10.1038/s41593-024-01576-9 PMC10917679 38388736

[92] Zenaro E, Piacentino G, Constantin G. The blood-brain barrier in Alzheimer’s disease. Neurobiol Dis. 2017;107:41–56. 27425887 10.1016/j.nbd.2016.07.007 PMC5600438

[93] Long H, Zhu W, Wei L, Zhao J. Iron homeostasis imbalance and ferroptosis in brain diseases. MedComm (2020). 2023;4(4):e298. 10.1002/mco2.298 PMC10292684 37377861

[94] Sofroniew MV. Astrocyte reactivity: subtypes, States, and functions in CNS innate immunity. Trends Immunol. 2020;41(9):758–70. 32819810 10.1016/j.it.2020.07.004 PMC7484257

[95] Fernández-Castañeda A, Lu P, Geraghty AC, Song E, Lee MH, Wood J, et al. Mild respiratory COVID can cause multi-lineage neural cell and myelin dysregulation. Cell. 2022;185(14):2452–e246816. 35768006 10.1016/j.cell.2022.06.008 PMC9189143

[96] Huang S, Zhou Z, Yang D, Zhao W, Zeng M, Xie X, et al. Persistent white matter changes in recovered COVID-19 patients at the 1-year follow-up. Brain. 2022;145(5):1830–8. 34918020 10.1093/brain/awab435 PMC8754808

[97] Huang S, Zhou X, Zhao W, Du Y, Yang D, Huang Y, et al. Dynamic white matter changes in recovered COVID-19 patients: a two-year follow-up study. Theranostics. 2023;13(2):724–35. 36632218 10.7150/thno.79902 PMC9830428

[98] Blanchard JW, Akay LA, Davila-Velderrain J, von Maydell D, Mathys H, Davidson SM, et al. APOE4 impairs myelination via cholesterol dysregulation in oligodendrocytes. Nature. 2022;611(7937):769–79. 36385529 10.1038/s41586-022-05439-w PMC9870060

[99] Zhao MM, Yang WL, Yang FY, Zhang L, Huang WJ, Hou W, et al. Cathepsin L plays a key role in SARS-CoV-2 infection in humans and humanized mice and is a promising target for new drug development. Sig Transduct Target Ther. 2021;6(1):1–12. 10.1038/s41392-021-00558-8 PMC7997800 33774649

[100] Mabrey FL, Morrell ED, Wurfel MM. TLRs in COVID-19: how they drive immunopathology and the rationale for modulation. Innate Immun. 2021;27(7–8):503–13. 34806446 10.1177/17534259211051364 PMC8762091

[101] Fontes-Dantas FL, Fernandes GG, Gutman EG, De Lima EV, Antonio LS, Hammerle MB, et al. SARS-CoV-2 spike protein induces TLR4-mediated long-term cognitive dysfunction recapitulating post-COVID-19 syndrome in mice. Cell Rep. 2023;42(3):112189. 36857178 10.1016/j.celrep.2023.112189 PMC9935273

[102] Song Y, Jiang W, Afridi SK, Wang T, Zhu F, Xu H et al. Astrocyte-derived CHI3L1 signaling impairs neurogenesis and cognition in the demyelinated hippocampus. Cell Reports [Internet]. 2024 May 28 [cited 2024 Jul 2];43(5). https://www.cell.com/cell-reports/abstract/S2211-1247(24)00554-0 10.1016/j.celrep.2024.114226 38733586

[103] CHI3L1 signaling impairs hippocampal neurogenesis. and cognitive function in autoimmune-mediated neuroinflammation | Science Advances [Internet]. [cited 2024 Jul 2]. https://www.science.org/doi/10.1126/sciadv.adg8148 10.1126/sciadv.adg8148 PMC10530095 37756391

[104] Zeng X, Cheung SKK, Shi M, Or PMY, Li Z, Liu JYH, et al. Astrocyte-specific knockout of YKL-40/Chi3l1 reduces Aβ burden and restores memory functions in 5xFAD mice. J Neuroinflamm. 2023;20(1):290. 10.1186/s12974-023-02970-z PMC10693711 38042775

